# *Ips typographus* vision system: a comprehensive study

**DOI:** 10.1007/s00359-024-01717-2

**Published:** 2024-09-27

**Authors:** Giuseppe Morgante, Gregor Belušič, Marko Ilić, Aleš Škorjanc, Enrico Negrisolo, Andrea Battisti

**Affiliations:** 1 Department DAFNAE, University of Padova, Viale dell’Università, 16, Legnaro, Italy; 2 Department BCA, University of Padova, Viale dell’Università, 16, Legnaro, Italy; 3 Department of Biology, University of Ljubljana, Večna pot 111, Ljubljana, Slovenia

**Keywords:** Bark beetle, Scolytinae, Light, Electroretinography, Opsin, Behaviour

## Abstract

Aggressive bark beetle species such as the Eurasian spruce bark beetle *Ips typographus* play a fundamental role in forest ecosystems but can also lead to extensive forest mortality and massive economic damage during outbreaks. Currently *I. typographus*’ eyes, visual perception of the world and recognition of specific targets like host plants are understudied topics. Studying its visual sense can open the way to novel efficient monitoring and management methods, particularly important in avoiding the switch from an endemic to an epidemic condition. In addition, the integration of visual cues in trapping systems may offer new opportunities for surveillance. Vision in *I. typographus* was investigated by means of morphological analysis, electroretinography (ERG), molecular analysis of opsin genes and behavioural tests. ERG has revealed that the compound eyes contain two classes of photoreceptors, maximally sensitive to UV and green at 370 and 530 nm, respectively. The result was further supported by the identification of two relevant opsin genes. Finally, the innate wavelength sensitivity was tested in a Y-maze. *Ips typographus* consistently preferred UV over non-UV (VIS) light, irrespective of their intensity ratios, but preferred high over low intensity VIS light, consistent with a UV-VIS dichromatic visual system. Overall, the results may open the way to better understand the navigation pattern in tree canopies and the host selection processes of this ecologically and economically important beetle species.

**Supplementary Information:**

The online version contains supplementary material available at 10.1007/s00359-024-01717-2.

## Introduction

Colour vision is regarded as the ability of an animal to analyse the spectral composition of light. It is the result of animal evolutionary adaptations to their environment, which ultimately leads to specific and unique, visually mediated behaviours (van der Kooi et al. 2021; Cronin et al. 2014; Dyer et al. 2012). To cope with the different ecological demands, insects have evolved very diverse sets of spectral photoreceptors. Yet, many insects from different orders retain the basic, ancestral set, based upon UV, blue and green-sensitive photoreceptors (Guignard et al., 2022). Beetles, however, represent an order with a documented evolutionary loss of blue opsin, which was in some clades secondarily substituted with a duplicated UV opsin (Jackowska et al. 2007; Sharkey et al. 2017).

Colour vision has been investigated in a few species of insect pests, alone (e.g., Lopez-Reyes et al. 2022) or in combination with other cues e.g. semiochemical and semiophysical (Nieri et al. 2022), yet the field remains largely unexplored. This is also the case in the Eurasian spruce bark beetle *Ips typographus* (Coleoptera, Curculionidae, Scolytinae), an insect pest that exploits the bark and the phloem of Norway spruce (*Picea abies*) for breeding, and is able to kill trees after a number of abiotic or biotic disturbances (Wermelinger 2004). Colonization starts with males looking for susceptible trees and, once having found a suitable one, attracting the conspecifics, both males and females using aggregation and sexual pheromones to generate a mass attack (Wermelinger 2004). Host recognition in *I. typographus* is complex and includes pre- and post-landing detection of chemical cues released by the tree (Lehmanski et al. 2023).

Little is known on the role of the visual system in host recognition by *I. typographus*. Bark beetles may use higher order visual features to identify the host plant. For instance, the southern pine beetle, *Dendroctonus frontalis*, is more attracted by dark coloured stems and vertical silhouettes (Strom et al. 1999) while Goyer et al. (2004) showed that American *Ips* spp. associated with pines discriminated not only differently coloured trap logs (black, white, unpainted), but also different log orientations (horizontal, vertical). Field experiments conducted with different species of bark beetles associated with conifers showed they prefer white colour (Hilker 1984; Sowinska 1993) or UV light (Pawson et al. 2009). The first physiological exploration of the visual sense in bark beetles was done by Groberman and Borden (1982) on *Ips paraconfusus*, for which they tested the spectral sensitivity and thus the ability of recognizing specific wavelengths of light using the electroretinography (ERG) technique and identified sensitivity peaks at 450 nm and 530 nm. Those results were later interpreted as the ability of bark beetles to use colour to avoid nonhost trees (Campbell and Borden 2006a, 2009). Considering the interdisciplinary studies conducted so far, Lehmanski et al. (2023) concluded that the visual capacity of *I. typographus* and other bark beetles seems to be rather limited and that a combination of visual and olfactory cues may serve as a stimulus for effective host recognition.

The objective of this study was to explore the vision of *I. typographus* and its sensitivity to different wavelengths. First, the spectral sensitivity of the eyes was recorded using electroretinography (ERG), measuring the retina’s response to light stimuli at different wavelengths. Second, the genome was explored for vision-related genes (opsins), as a validation of the findings given by the ERG. Third, the individual responses to specific wavelengths were tested through behavioural assays, by observing how males and females interact with specific wavelengths and intensities. Such findings may provide indications for the integration of visual cues in host recognition by *I. typographus*, indicating potential avenues for surveillance (e.g. more efficient trapping systems) and management strategies of the populations.

## Materials and methods

### Model system and beetle collection

Bark samples measuring about 20x30 cm and containing overwintering adults were collected in winter (November and December 2022 and March 2023) from selected infested trees in an outbreak area of the southern Alps (Veneto, Gares, 46.307° N 11.866° E; 46.330° N 11.890° E). Five to six bark samples were placed in ventilated plastic boxes (37x26 cm) and stored in laboratory refrigerators at 4°C with no light, thus maintaining hibernation. Beetles used to run ERG were kept outside of refrigerators and maintained on an artificial diet (Kandasamy et al. 2023). The medium was autoclaved and placed in 2 ml Eppendorf tubes fully filled. Caps were perforated to allow air circulation.

### Eye morphology

To obtain images of the eyes, heads were firstly isolated under a stereo microscope (Zeiss mod. Stemi 305) and antennae were removed using a scalpel. Subsequently, isolated heads were inserted into a SEM microscope (Hitachi mod. TM-1000) and observed at 400x magnification. This magnification allowed to include the whole eye in the image. To characterize eye morphology, a total of 50 individuals (26 females, 24 males) were used.

Four axes across the eye were virtually overlapped, one longitudinal on the maximum length and three transversal on the largest point of upper, middle, and lower portions. The length of each axis was measured and the number of ommatidia crossed by each axis was counted. The width of ommatidia was calculated dividing the length of the axis by the number of ommatidia crossed by the axis. Areas of single ommatidia were calculated drawing the perimeter on 10 randomly chosen ommatidia in each portion of the eye (upper, medium, lower). Finally, on 13 eyes of randomly selected individuals (6 females, 7 males), all ommatidia were counted.

Eyes images were analysed using IC Measure software (The Imaging Source, LLC, Germany). Statistical analyses were conducted using the R studio software (R Core Team 2021). ANOVA test and t test were used to evaluate significant differences. The significance level used was alpha = 0.05.

For micro-computed tomography (micro-CT), isolated heads were fixed in 70% ethanol and stained with 1% PTA (phosphotungstic acid dissolved in 70% ethanol) for 2 days. After incubation samples were washed several times in 70% ethanol and transferred into heat-sealed pipette tips filled with 70% ethanol. Scans were performed with Neoscan N80 microtomograph (Neoscan, Mechelen, Belgium) at 50 kV, 80 µA and 0.7 μm resolution. The sample was rotated in 0.2° steps over a total of 180°, averaging 3 frames for each step. Virtual cross sections were reconstructed from 988 projection images with Neoscan 80 software (version 2.2.4) (Neoscan, Belgium). A total of 2800 sections were imported into Dragonfly software (version 2022.2.0.1399) (ORS Inc., Mississauga, Ontario, Canada) for 3D reconstruction and obtaining projections in arbitrary planes.

### Spectral sensitivity measurements

Measurements were conducted in March 2023 at the University of Ljubljana, Department of Biology, on beetles obtained from the samples described above.

To test their spectral sensitivity, beetles were immobilized with beeswax with the recorded compound eye positioned in the rotation centre of a goniometric stage, which also carried the micromanipulator (Sensapex, Finland). The reference electrode was a 50 μm diameter Ag wire coated with AgCl, inserted into the abdomen, while the recording electrode was a blunt glass micropipette, pulled on a laser puller (P-2000, Sutter, USA), filled with insect saline solution. ERG signals were amplified (SEC-10LX, Npi, Germany), band-pass filtered (0.1–50 Hz; Cyberamp, Molecular Devices, USA), digitized with an AD/DA converter (Micro1401 mkII, Cambridge Electronic Design, Ltd., UK), and stored and analysed with WinWCP, version 5.5.4 (John Dempster, University of Strathclyde, Glasgow, UK). The beetles were oriented with respect to the stimulating beam to yield a maximal ERG response.

Stimulation was provided with two coaxial light sources: an LED array (Belušič et al. 2016) and a xenon arc lamp (XBO, Osram, Germany), filtered with a monochromator (B&M, Germany) and intensity adjusted with a motorized graded neutral density filter (Thorlabs, Germany). Each source could be used for the creation of monochromatic flashes or for constant selective adaptation with monochromatic light. Both light sources were calibrated with a Flame spectroradiometer (Ocean optics, USA) to yield equal photon flux (max. 1∙10^15^ quanta cm^− 2^ s^− 1^) at every wavelength.

### Bioinformatic analyses

The full-length DNA sequences encoding for long wavelength (LW) and ultraviolet absorbing (UV) opsins belonging to beetles of the superfamily Curculionoidea were downloaded from Genbank together with selected full-length DNA encoding LW and UV opsins sequences from beetles belonging to Cucujoidea and Chrysomeloidea (Sharkey et al. 2021). These superfamilies are the most closely related to Curculionoidea in the current reference phylogeny for Coleoptera (Sharkey et al. 2021). The last version available of the genome of *I. typographus* (Powell et al. 2021) was analysed to identify the LW and UV opsins encoded in its sequence, because these data are not available in GenBank. The full set of opsin sequences analysed in present paper is listed in Table S1 - Supplementary document, where we provide a detailed description of the many molecular analyses that we have done for present paper.

The physical and chemical parameters for the opsins of *I. typographus* were computed with the ProtParam program available at the the Expasy Swiss Bioinformatics Resource Portal (Duvaud et al. 2021). The 3D-structures of the LW opsin and UV opsins of *I. typographus* were determined through a homology modelling strategy implemented in the SWISS-MODEL Workspace available at https://www.expasy.org.

Three set of opsins CURC-set, LWs-set and UVs-set were created (Table S2). The DNA sequences were translated into the proteins. These latter were aligned with the software MAFFT (L-INS-I, option) (Katoh et al. 2005). Successively, each set of the protein-coding sequences was aligned on the TranslatorX server using as template the alignment obtained for the encoded proteins (Abascal et al. 2010). Once downloaded from the TranslatorX server, the DNA multiple alignments were uploaded in MEGA X software (Kumar et al. 2018) to be saved in various subsets analysed in our paper. The compositional heterogeneity of a multiple alignment is a major source of misleading phylogenetic outputs (Kück et al. 2014). The level of compositional heterogeneity was tested with AliGROOVE (Kück et al. 2014). The amount of phylogenetic signal present in the 18 multiple alignments, analysed in this paper, was evaluated studying the distribution of the pairwise distances (based on the best fitting evolutionary models) using boxplots (Fig. S3, S4, S5, S6, S7, S8). Phylogenetic analyses, based on the maximum likelihood method, were performed with the software IQ-TREE 2.3.4 (Minh et al. 2020). The best fitting evolutionary models of the different data sets were selected using the ModelFinder algorithm implemented in IQ-TREE (Kalyaanamoorthy et al. 2017). Partitioned analysis implemented in IQ-TREE was applied to the DNA multiple alignments (Chernomor et al. 2016). Statistical support to the tree topologies were assessed by computing 10,000 replicates of ultrafast bootstrap (UFBoot) (Hoang et al. 2018) and 1000 replicates of the SH-like approximate likelihood ratio test (SH-alrt) (Guindon et al. 2010).

### Behavioural experiments

Innate wavelength preferences of the beetles were tested in a Y-maze (Fig. S11). The maze was constructed over a customized aluminium support built with Makerbeam (Utrecht, Netherlands) out of black-opaque honeycomb polypropylene sheets (thickness 2 mm). Sheets were glued to build a squared open-top corridor where beetles were able to walk on both the bottom and the sides. The length of the initial corridor was 12 cm. A sliding door made of polypropylene sheet was inserted at the base of the corridor to isolate the beetle before the starting of the test. The arms were 8 cm long, with an angle of 120°. Each arm ended with a 3 cm window made of a non-fluorescent diffusor (lens cleaning tissues 105 W, Whatman, USA) inserted into a frame of the same polypropylene sheet, preventing the beetle to walk further. The light sources were located at a distance of 8 cm from the arm windows and consisted of LEDs selected based on the wavelengths to which the beetles responded in the physiological tests. The distance was chosen to concentrate the light beam on the light diffusor window.

Light sources used for the tests were the UV LED (SMB1N-375 V) and the green LED (SMB1N-525 V) by Roithner Lasertechnik GmbH (Vienna, Austria). Conditions where the LEDs would appear subjectively equally bright to the beetles were created, i.e., the LED output would evoke approximately equal depolarization in both spectral channels. Then, the intensity could be adjusted to create subjectively dimmer or brighter UV or green light, to test whether the beetles would orient on the basis of chromatic, not intensity cues. It was assumed that the photoreceptor gain has evolved so that both spectral channels are about equally depolarized when exposed to daylight illuminant (Chittka et al. 1994). To fulfil these assumptions, the LED photon flux was computed based on the spectral sensitivity recordings and the standard daylight irradiance spectrum (CIED65). First, the photon catch *N* of *I. typographus* UV and green-sensitive photoreceptors was calculated as

$$\:{N}_{i}=\underset{300}{\overset{700}{\int\:}}{R}_{i}\left(\lambda\:\right)I\left(\lambda\:\right)d\lambda\:$$

where *R*_i_ is the normalized spectral sensitivity *R* of the photoreceptor class i, *I* is the normalized CIED65 irradiance spectrum and the wavelength range is 300–700 nm. The relative quantum catch for each of the two receptor channels was obtained by dividing the quantum catch of an individual channel by the sum of quantum catches in both channels. It was calculated that under daylight, the green-sensitive photoreceptors catch four times more photons than UV-sensitive photoreceptors and assumed that the phototransduction gain (i.e. membrane voltage change per absorbed quantum) is 4 times higher in the UV photoreceptors than in the green photoreceptors. Consequently, to imitate the receptor excitation under natural conditions, the LEDs were set with a quantal flux ratio of 1:4 for UV and green light respectively. Based on the above mentioned phototransduction gain, to test the chromatic preference at different beetle-subjective stimulus intensities, quantal flux ratios of 1:1 and 1:16 for UV and green light respectively were used. In particular, UV being experimentally brighter (1:1), equal (1:4) and dimmer (1:16), in relation with the green light. The LEDs were powered by a lab power supply powering pickobuck LED drivers (mod. COM-13705 ROHS, Sparkfun), controlled with a Teensy microcontroller (NXP microcontrollers, The Netherlands). LED intensity was adjusted with pulse width modulation at 1 kHz, well beyond the flicker fusion frequency of any animal (Autrum 1958). The illumination system was calibrated with a Flame spectroradiometer (Ocean optics, USA) so that the maximal light output of the green LED was 10^13^ quanta s^− 1^ cm^− 2^. All tests were performed in a dark room illuminated with ambient red light (20 W tungsten light bulb, filtered with a KODAK safelight-filter mod. GBX-2, mounted directly on the maze arm), which was shown to have no effect on beetle behaviour in preliminary tests.

Tests were run in July 2023 on beetles obtained from the bark samples described above and gradually taken to laboratory room temperature (26 –29 °C), as they were leaving the overwintering conditions. Only beetles that showed a standard activity of movement were selected for the tests 2 h after they reached the room temperature under laboratory light conditions. The experiments were run between 11:30 and 15:00. Each individual was given a maximum of 10 min for the test. Beetles were first placed individually in the dark part of the basal corridor for 1 min. Once the sliding door was open, the test started, and notes of movements were taken during the experiment. A clear final choice was declared after the individual spent at least 1 min continuously at the end of the same arm of the maze as already done in behavioural choice experiment by Netherer et al. (2022). The measure considered for all the experiments is the time (expressed in seconds) used by beetle to reach the arm. The tests included: (i) green light vs. no light, (ii) UV light vs. no light, (iii) UV vs. green light with 1:4 quantal flux ratio, (iv) UV vs. green light with 1:1 quantal flux ratio, (v) UV vs. green light with 1:16 quantal flux ratio. The light sources were randomly alternated between the two arms for each individual tested. Only naïve beetles were used, for a total of 10 females and 10 males in each test.

In June 2024, to test the discrimination of wavelength vs. brightness cues in the spectral band where colour vision capability was not expected (420–600 nm), the same protocol was used with further 10 beetles (5 females, 5 males collected from the field) per each colour (blue (470 nm) and yellow (590 nm)). Tests included green (530 nm) vs. blue and yellow using a 1:4 ratio.

### Data analysis

In the tests light versus no light, all the individuals chose the lit arm (either green or UV), therefore a linear model was employed to assess the time needed to reach the light source (response variable) in relation to the light colour (categorical variable with two levels: green or UV), the sex (categorical variable with two levels: female or male), and their interaction. The response variable was log-transformed to improve model linearity.

In the tests green versus UV light, a Pearson chi-square test was used to test the preference toward a light source for each quantal flux ratio. Since in all tests the preference toward UV light was between 95 and 100%, a linear model was employed to assess the time needed to reach the UV light source in relation to the sex, the quantal flux ratio (categorical variable with three levels of UV to green ratio, i.e., 1:1, 1:4 or 1:16), and their interaction.

Pairwise multiple comparisons were performed using adjusted p-values with Tukey correction. The significance level used was alpha = 0.05. Statistical analyses were performed using the R software (R Core Team 2021).

## Results

### Eye morphology

**Fig. 1:**
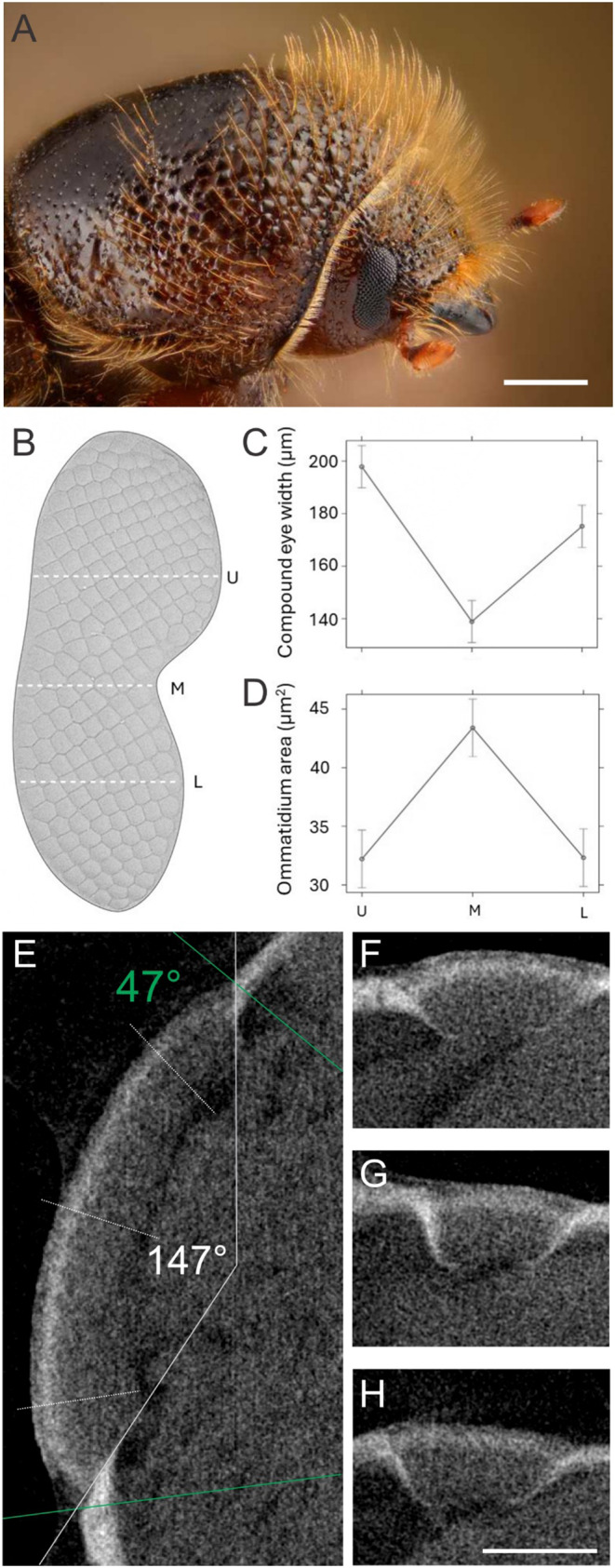
Morphology of *Ips typographus* eyes. **A** Macro photo of the pronotum and the head (Gilles San Martin). **B** Ommatidia of the left eye, segmented from a scanning electron micrographs; U, M, L, upper, middle, lower section of the eye. **C** Width of the compound eye and **D** ommatidial area at the sections U, M, L. **E** Micro-CT scan along the dorso-ventral plane of the male eye; dotted lines indicate horizontal planes, at which the scans in F-H were obtained; white lines indicate the estimated viewing angle of the eye (147°), green lines indicate the estimated angle of the head, occupied by the eye (47°). **F**, **G**, **H** Micro-CT scans along the dotted lines in E. Scale bars: A, 500 μm, H, 100 μm

The visual system of *Ips typographus* is composed of a pair of small compound eyes (Fig. 1). Each eye is composed on average of 212 ommatidia for females (SE ± 6.67) and 210 (SE ± 6.6) for males. The eye length along the dorso-ventral axis is ~ 500 μm, the width is ~ 200 μm dorsally, ~ 140 μm centrally and ~ 180 μm ventrally. The facet surface area varies between 32 µm^2^ at the dorsal and ventral extremes and 42 µm^2^ centrally. The head cross-section in the coronal plane is almost ideally cylindrical, and the angular size of each eye in the dorso-ventral direction is about 47°. However, the viewing angle seems to be larger, as the ommatidia are highly skewed. The estimated viewing angle along the dorso-ventral axis is ~ 147°, yielding an inter-ommatidial angle Δφ ~ 6°. No significant differences were found between males and females and between left and right eyes.

### Spectral sensitivity

**Fig. 2:**
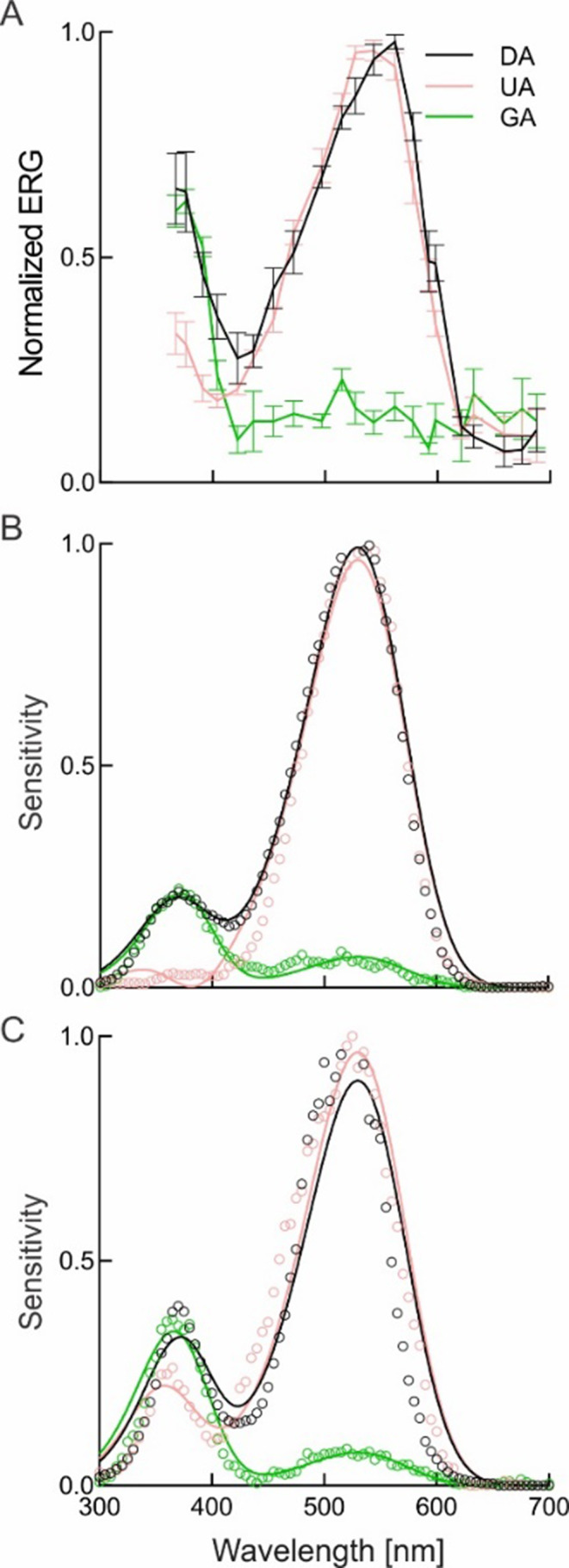
Spectral sensitivity (SS) of *Ips typographus*, measured with ERG and selective chromatic adaptation; DA, dark-adapted; UA, UV-adapted; GA, green-adapted. **A**, SS measured with LED array (range 365–700 nm, 15 nm interval) and adaptation from the monochromator at 360 and 530 nm (*n* = 4, mean ± SEM, both sexes merged). **B**, **C**, SS measured with the monochromator (range 300–700 nm, 5 nm interval) and adaptation from LEDs at 375 and 542 nm. B, female (*n* = 1); C, male (*n* = 1). Smooth curves in B, C are rhodopsin absorption templates with amplitudes as free parameters and fixed absorption peaks (UV rhodopsin, λ_max_ = 370 nm; green rhodopsin, λ_max_ = 530 nm)

Electroretinography recordings showed two peaks of spectral sensitivity, one in the UV and one in the green part of the spectrum (Fig. 2A). Chromatic adaptation with UV and green light selectively suppressed sensitivity in the corresponding spectral regions in both female (Fig. 2B) and male (Fig. 2C) beetles. We concluded that the retina of *I. typographus* thus contains two classes of photoreceptors, the UV-sensitive with peak at 370 nm and green-sensitive with peak at 530 nm.

### Opsins

The genome search has revealed two opsin encoding genes: a long wavelength absorbing opsin (Ityp07182 LW opsin) containing 377 amino acids with an estimated molecular weight of 41976.29 Daltons and a theoretical pI = 8.68 and an ultraviolet absorbing opsin (Ityp12360 UV opsin) containing 373 amino acidic residues for a total molecular weight of 42064.25 Daltons and a theoretical pI = 8.13. The tertiary (3D) structures inferred for both Ityp07182 LW opsin and Ityp12360 UV opsin contain 14 α-helices and 2/4 β-sheets and exhibit a high structural similarity (Fig. S2). Twenty-three full-length opsins were identified among beetles of the superfamily Curculionoidea (Table S1). The multiple alignment of these opsins is provided in the Fig. S3. Pairwise distances, based on the best fitting evolutionary models, reveal a strong conservation among the opsins of the same type (LW opsins; average-distance = 0.189 ± 0.122. UV opsins, average-distance = 0.317 ± 0.105), with the LW opsins being more conserved than UV opsins (Fig. S7B). On the opposite, the distances clearly exceed one (average-distance = 2.889 ± 0.199) when they are computed for pairs of paralogous sequences (i.e. LW vs. UV; Fig. S7B).

**Fig. 3:**
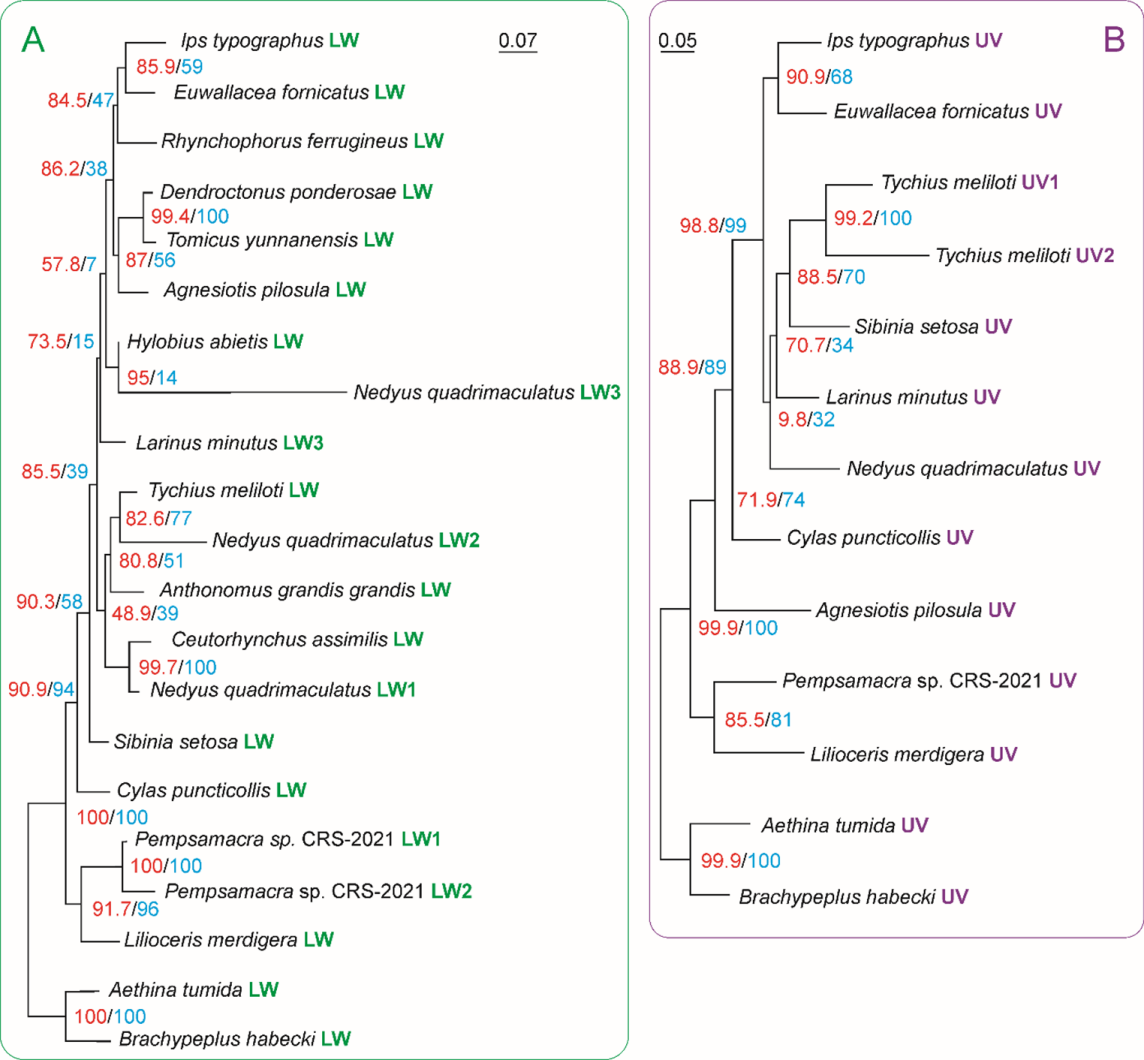
Phylogenetic trees depicting the relationships among LW and UV opsins. (**A**) Maximum likelihood phylogram (-log = 5144.9497) obtained from LWss-set.p12 with IQ-TREE by applying the best fitting evolutionary model: TIM3e + G4:part1 (i.e. first positions), TVM + F + I + R2:part2 (i.e. second positions). (**B**) Maximum likelihood phylogram (-log = 4765.3053) obtained from UVs-set.p12 with IQ-TREE by applying the best fitting evolutionary model: TIM2e + G4: part1 (i.e. first positions), TPM3u + F + R2: part2 (i.e. second positions). Scale bars represent number of substitutions per site. UFBoot/SH-alrt expressed in percentage

In the multiple alignment the amino acids shared by the whole set of LW and UV opsins are located mainly in the long transmembrane α-helices (Fig. S3). Phylogenetic analyses were performed on LWs-set.p12 and UVs-set.p12 containing only the first and second positions of codons of orthologous sequences (Fig. 3 and S3-S5). The phylogenetic relationships among the opsin sequences in both trees are in good agreement with the taxonomic placement of the species from what they were obtained with some exceptions (e.g. the placement of *Agnesiotis pilosula* LW). In the tree obtained from LWs-set.p12 most of the nodes and branches received statistical corroboration (Fig. 3A). The LW sequence of *I. typographus* is grouped with the sequence of *Euwallacea fornicatus* LW and *Rhynchophorus ferrugineus* LW. Most of nodes and branched of the tree obtainded from obtained from UVs-set.p12 multiple alignment receive statistical support. In this tree the UV sequence of *I. typographus* is clustered with the UV sequence of *E. fornicatus* and this relationship receive statistical corroboration. Like in the LW tree, there is good agreement with opsins relationships and taxonomic placement of the species.

### Behavioural experiments

**Fig. 4:**
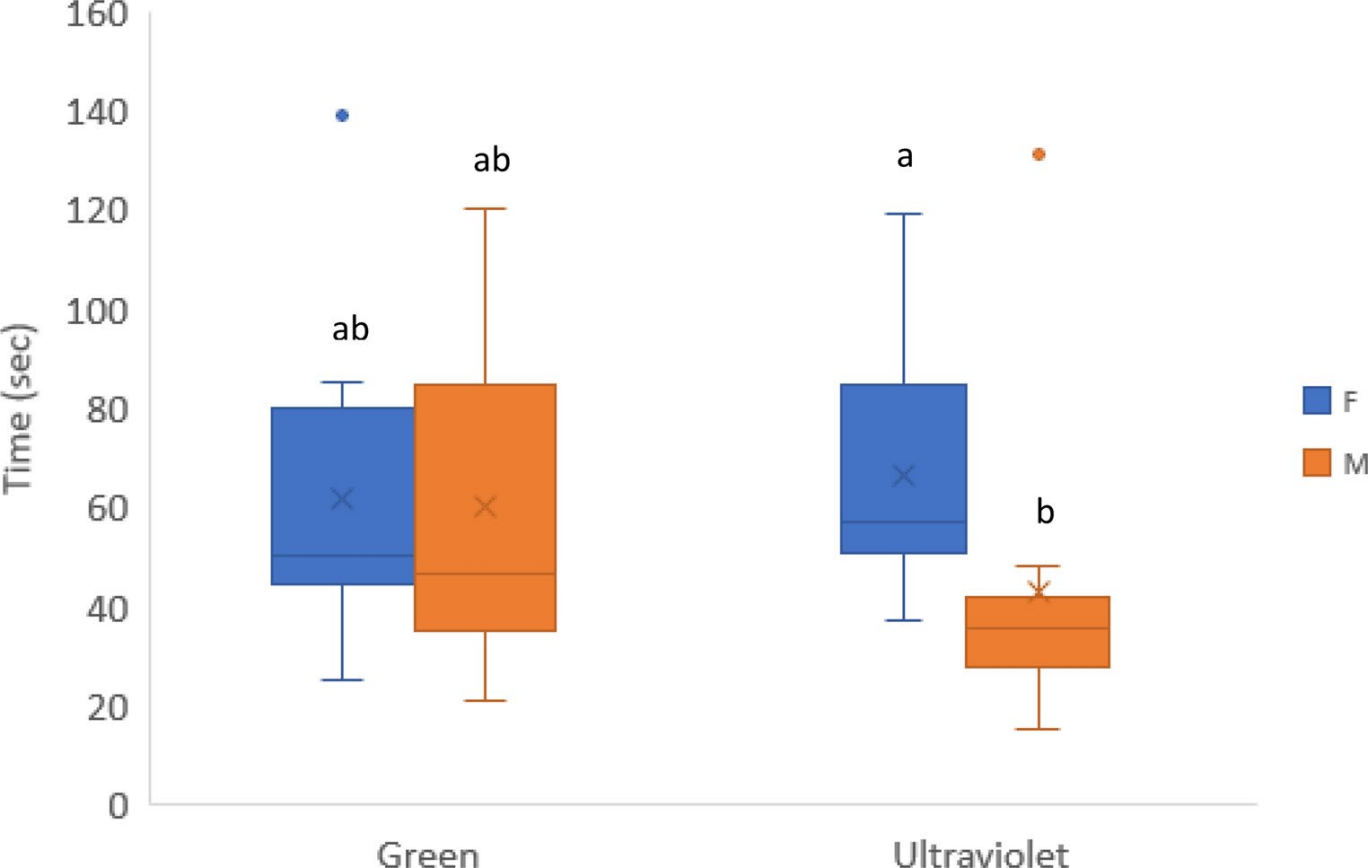
Time needed to reach the green or UV light source for females and males in the light vs. no light test (none of the individuals reached the non-illuminated arm). Letters a, b indicate significant differences (*p* < 0.05) in the pairwise comparisons between females and males and different light sources. Values on y-axis were back transformed from the log-scale used for testing

When green and UV were tested against no light, all beetles chose the illuminated arm, either with green light (time to reach the end of the arm: 60.01 s (SE ± 10.26) for males and 61.8 s (SE ± 10.20) for females) or with UV light (time to reach the end of the arm: 42.6 s (SE ± 10.28) for males and 81.8 s (SE ± 17.42) for females). The interaction light × sex in the time to reach the illuminated arm was close to significance (*p* = 0.072), with a tendency for males to respond faster than females when challenged with UV light (Fig. 4). The behaviour of the beetles was also affected by the wavelength of light. Under green light illumination, beetles slowly walked their way towards the light source, exploring the environment and, occasionally, turning toward the arm of the maze with no light source. Finally, when the light source was reached, they climbed the frame until its edge was reached, walking back on the illuminated area. Under UV illumination, males walked straight and quickly toward the light source while females often stopped on their way, exploring the surroundings but never moving away from the light beam. The dark arm of the maze was never visited by both sexes. When the frame was reached, only few individuals climbed to the transparent part, most beetles remained at the bottom of it, trying to dig into the corners.

**Fig. 5:**
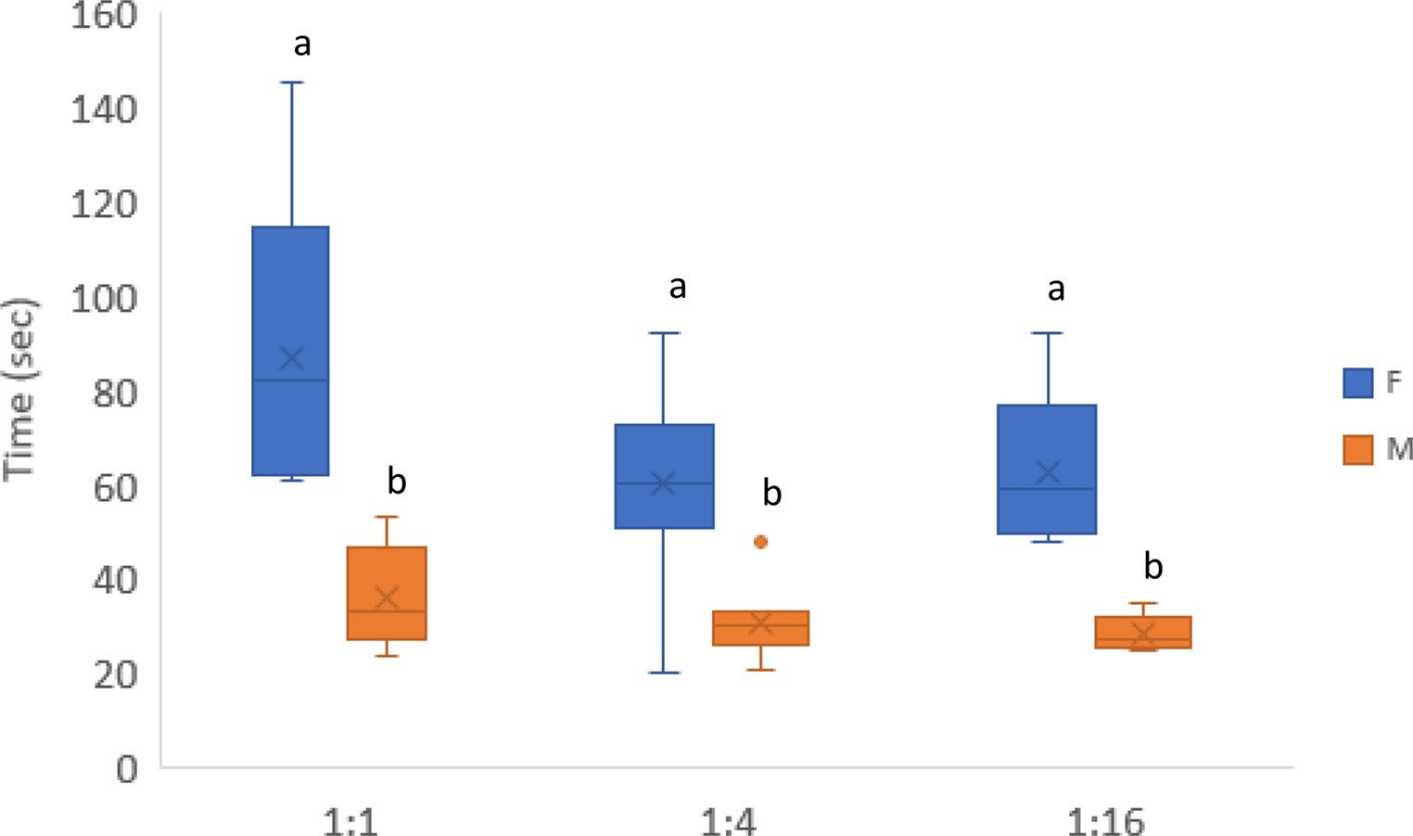
Time needed to reach the UV light source for females and males in the three quantal flux ratio tests. For each quantal flux ratio test, different letters indicate significant differences (*p* < 0.05) in the pairwise comparisons between females and males. Values on y-axis were back transformed from the log-scale. Letters **a**, **b** indicate significant differences (*p* < 0.05) in the pairwise comparisons between females and males and different light sources

When UV was tested against green light with 1:4 quantal flux ratio, most beetles (19/20, 95%) chose the UV light, with the exception of one female. The time needed by males to reach the light source (30.5 s, SE ± 2.29) was significantly shorter compared with females (75.3 s, SE ± 16.32) (*p* < 0.001). In tests performed with quantal flux ratio 1:1 between UV and green light, the UV light was chosen by all beetles (20/20, 100%). Again, a significant difference to reach the light source was found between males (36 s, SE ± 4.97) and females (87 s, SE ± 15.25) (*p* < 0.001). With the quantal flux ratio 1:16 between UV and green, the same clear preference towards UV light (20/20, 100%) was maintained. The time taken to reach the light source was 28.4 s (SE ± 1.78) for males and 62.4 s (SE ± 7.83) for females, with significant difference (*p* < 0.001). The interaction quantal flux ratio × sex in the time to reach the illuminated arm was not significant (*p* = 0.877) (Fig. 5).

**Fig. 6:**
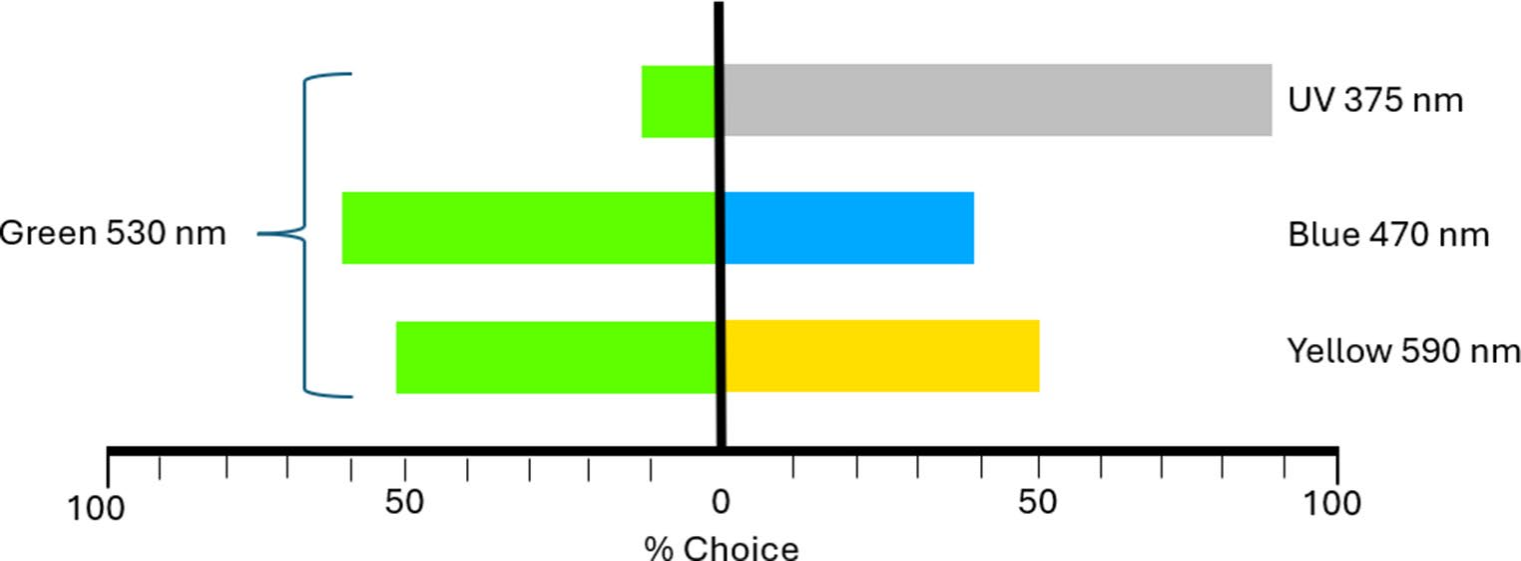
Summary of the choice experiments of beetles to different light sources. *indicates significant differences

When the beetles were allowed to choose the different colours in the non-UV part of the spectrum (blue, green and yellow) at subjectively equal brightness levels, they showed no wavelength preference (*p* = 0.51) (Fig. 6). The time taken to reach the chosen light source was not different between sexes and similar to the one observed for green light when tested against UV light with post-overwintering individuals. Overall, the beetles did not prefer any wavelength out of the UV part of the spectrum (*p* = 0.014) (Fig. 6).

## Discussion

The bark beetle *I. typographus* has compound eyes with a low resolution and a dichromatic vision system, based on UV (370 nm) and green (530 nm) photoreceptors, typically found in insects that do not visit flowers (Sharkey et al. 2017). The similar visual system of *Tribolium* beetles is based upon UV and green photoreceptors that coexpress the two opsins (Jackowska et al. 2007), but our ERG method was insufficiently precise to show a putative co-expression in the two receptor classes. The beetles are innately attracted towards UV light sources, which are preferred over non-UV light, irrespective of their intensity and the state of the adults immediately prior to ERG. However, they do not show any wavelength preference in the non-UV part of the spectrum, which is consistent with the finding of a single receptor type and a single opsin in the non-UV part. Thus, we can conclude that the beetles have a simple, dichromatic colour system, which can be used to discriminate UV from non-UV cues and guide the locomoting beetles.

The sensitivity of *I. typographus* to UV light was already mentioned in pioneer studies by Hilker (1984) but never explored at physiological, molecular, and behavioural level. Using selective adaptation, it was not possible to detect a specific peak in the spectral sensitivity in the blue part of the spectrum. Consistent with the electrophysiological and behavioural results, the genome does not appear to contain a blue opsin. These results are very different from those previously reported for the closely related beetles *Ips paraconfusus* and *Dendroctonus pseudotsugae*, which were reported to be maximally sensitive to blue light (Groberman and Borden 1982). In light of the new findings related to physiology and genetics, we suspect that the reported blue sensitivity was erroneous, possibly due to data misinterpretation. The sensitivity to UV light was also supported by behavioural tests, where an instant recognition and subsequent response were observed when individuals were exposed to different quantal flux ratios. Both males and females showed a distinct preference for the UV, regardless of the ratio, although males always had a faster response, as already observed for the response to colours by *Dendroctonus* bark beetles in North America (Campbell and Borden 2006b). The results complement what is known for bark and other beetles in relation to the responses to light stimuli, based on a remarkable ability to evolve and adapt to environmental conditions (Sharkey et al. 2017).

The opsin proteins of *I. typographus* resulted also in good agreement, especially in the arrangement and size of the long transmembrane α-helices with the opsin structures, determined through crystallography, of the squid *Todarodes pacificus* (Murakami and Kouyama 2008) and of the jumping spider *Hasarius adansoni* (Varma et al. 2019). LW (green) and UV opsins exhibit a strong similarity in terms of predicted 3D-structure, not a surprising result provided that both are the protein constituents of rhodopsins. Conversely, they show a diverging evolution for what concerns the amino acid sequences as it can be appreciated by the inspection of their multiple alignment and the computation of their distances. The multiplication of the ancestral opsin into paralogous diverging genes and protein products is an old event in insects (Henze and Oakley 2015; Feuda et al. 2016). In our study, the divergent evolution that characterized also the LW and UV opsins of Curculionoidea is strongly supported by their very large pairwise distances (Fig. S7B). The substitution process was drastically slowed down within each opsin type, as proved by the high degree of sequence conservation (Fig. S3) and the small distances among orthologous sequences (Fig. S7-S9). A pivotal role in the reduction of sequence change was played by structural and functional constraints. Strongly favouring this hypothesis is that third positions of both the LW and UV opsin coding genes (Fig. S7-S9) have experienced multiple substitutions as proved by their very large distances. Most of these changes are synonymous i.e. they do not imply changes of amino acid. However, it should be kept in mind that the comparisons were done with opsins obtained from related beetles. Thus, phylogenetic relatedness could contribute partly to the sequence stability.

In the literature there is limited information on how bark beetles use visual cues during the host selection process. Campbell and Borden (2005, 2006a) supported the idea that *Dendroctonus ponderosae* is able to integrate and process both olfactory and visual cues at a relatively close distance from the host (≤ 2 m). At a larger range, it is generally accepted that olfactory cues are the main players (Lehmanski et al. 2023). Based on new results on UV sensitivity, it can be hypothesized that beetles just emerged from the overwintering can respond to UV during the initial flight dispersal phase (Forsse and Solbreck 1985). Diurnal insects have evolved distinct UV-sensitive spectral channels in the visual systems (Cronin and Bok 2016), allowing them to navigate under canopy through vegetation using patches of enhanced UV light acting as signatures of the open sky. The abundance of UV light is due to the Rayleigh scattering by molecules in the atmosphere, which is more efficient at shorter wavelengths, giving rise to the blue colour of the sky due to increasing scattering of blue and UV light (Cronin and Bok 2016). The sensitivity to green colour could help *I. typographus* in recognizing plants during the flight over the canopies (Forsse and Solbreck 1985), in association with odour sources. Lacking any sophisticated colour system, *I. typographus* can still identify host plants using brightness cues (i.e., dark canopies of conifers) detected by the green photoreceptors.

The shorter time spent by male beetles to reach the UV light source in both tests UV light vs. no light and UV vs. green light can be explained by the distinct roles that each sex plays in host colonization. Males of *I. typographus* act as pioneers, actively seeking out suitable plants in the landscape, a process generally driven by volatile compounds released by the trees (Lehmanski et al. 2023). The faster response of males to UV light may suggest they are more dependent than females to this type of cue, which could help them during navigation and search for suitable trees, while females are more dependent on olfactory cues. In the UV vs. green light assays, the only individual selecting the green light was indeed a female, suggesting the distinct light-related traits associated with sexes. Furthermore, different quantal flux ratio tests revealed a true chromatic preference and a constant different time response to light stimuli by both sexes.

## Conclusions

*Ips typographus* has a primitive, dichromatic visual system, which is nevertheless the basis for wavelength discrimination, independent of light intensity. The new knowledge acquired on the visual system of *I. typographus* may open the way to a thorough exploration of its role in host finding, especially when the various phases of the process will be investigated. We suggest that the visual cues used in trapping include dark silhouettes, resembling tree trunks, contrasted against a UV-enriched background. The research should take into account an accurate phylogenetic screening of opsins genes, a detailed histological analysis of the compound eye, testing flying beetle in wind tunnel and considering their mating status. The results may have practical implications, such as the development of more efficient trapping devices based on the integration of olfactory and visual cues.

## Electronic supplementary material

Below is the link to the electronic supplementary material.

Supplementary Material 1

Supplementary Material 2

## Data Availability

No datasets were generated or analysed during the current study.
