# Supplementary material for: Ips typographus vision system: a comprehensive study
Source: J Comp Physiol A Neuroethol Sens Neural Behav Physiol. 2024 Sep 27;211(1):101–12. doi: 10.1007/s00359-024-01717-2 (PMC11846742; doi:10.1007/s00359-024-01717-2)
Supplement: Supplementary file 1 — Supplementary Material 1 [file 359_2024_1717_MOESM1_ESM.pdf]

Supplementary Document

Molecular Evolution of the LW and UV opsins of *Ips typographus*

Premise

The main goals of our bioinformatic analyses were to get information on the structure and evolution of LW and UV opsins of *I. typographus*. In particular, our molecular evolution study was focused on the phylogenetic placement of the LW and UV opsins of *I. typographus* among the available orthologous counterparts known for the species of beetles of the superfamily Curculionoidea. The study of the evolution of the opsins for the whole order Coleoptera or more in general for the class Insecta, to remain within Arthropods, was not the focus of our work. Several papers are already available on this topic and the reader must consult them for further details (e.g. Henze and Oakley 2015; Fueda et al. 2016; Sharkey et al. 2017; Sharkey et al. 2021; Guignard et al. 2022; McCulloch et al. 2022).

Methods

Structural Bioinformatics

The physical and chemical parameters for the protein sequences of the LW and UV opsins of *I. typographus* were computed with the ProtParam program available at the the Expasy Swiss Bioinformatics Resource Portal (Duvaud et al. 2021). The 3D-structures of the LW and UV opsins of *I. typographus* were determined through a homology modelling strategy implemented in the SWISS-MODEL Workspace available at https://www.expasy.org.

Data set construction

Initially the full-length DNA sequences encoding for long wavelength (LW) and ultraviolet absorbing opsins (UV) belonging to beetles of the superfamily Curculionoidea were downloaded from Genbank following the work of Sharkey et al. (2021). These sequences were translated into proteins that were used to search for further opsins in GenBank through the BLASTP algorithm (Altschul et al. 1990). The last version available of the genome of *I. typographus* (Powell et al. 2021) was analysed to identify the LW and UV opsins encoded in its sequence, because these data are not available in GenBank. Finally were added to the data set selected full-length DNA encoding LW and UV opsins sequences from species of beetles belonging to the superfamiles Cucujoidea and Chrysomeloidea, which are the most closely related taxa to Curculionidea in the current reference phylogeny for Coleoptera (see Sharkey et al. 2021). The full set of opsin sequences analysed in present paper is listed in Table S1.

Table S1. List of full-length opsins of species of beetles of the superfamilies Cucujoidea, Chrysomeloidea, and Curculionidea, analysed in present paper

Family	Subfamily	species	Accession/code	acronym
Nitidulidae	Nitidulinae	*Aethina tumida* Murray, 1867	KY368241	**LW**
Nitidulidae	Nitidulinae	*Aethina tumida* Murray, 1867	XM_020009244	**UV**
Nitidulidae	Cillaeinae	*Brachypeplus habecki* Cline and Skelley, 2013	MW886077	**LW**
Nitidulidae	Cillaeinae	*Brachypeplus habecki* Cline and Skelley, 2013	MW886076	**UV**
Chrysomelidae	Criocerinae	*Lilioceris merdigera* (Linnaeus, 1758)	MW886030	**LW**
Chrysomelidae	Criocerinae	*Lilioceris merdigera* (Linnaeus, 1758)	MW886031	**UV**
Cerambycidae	Cerambycinae	*Pempsamacra* sp. CRS-2021	MW886045	**LW1**
Cerambycidae	Cerambycinae	*Pempsamacra* sp. CRS-2021	MW886043	**LW2**
Cerambycidae	Cerambycinae	*Pempsamacra* sp. CRS-2021	MW886046	**UV**
Belidae	Belinae	*Agnesiotis pilosula* Pascoe, 1870	MW886010	**LW**
Belidae	Belinae	*Agnesiotis pilosula* Pascoe, 1870	MW886009	**UV**
Brentidae	Cyladinae	*Cylas puncticollis* (Boheman 1883)	KY368289	**LW**
Brentidae	Cyladinae	*Cylas puncticollis* (Boheman 1883)	KY368290	**UV**
Curculionidae	Ceutorhynchinae	*Ceutorhynchus assimilis* (Paykull, 1792)	CAH1123441	**LW**
Curculionidae	Ceutorhynchinae	*Nedyus quadrimaculatus* (Linnaeus, 1758)	MW886037	**LW1**
Curculionidae	Ceutorhynchinae	*Nedyus quadrimaculatus* (Linnaeus, 1758)	MW886038	**LW2**
Curculionidae	Ceutorhynchinae	*Nedyus quadrimaculatus* (Linnaeus, 1758)	MW886039	**LW3**
Curculionidae	Ceutorhynchinae	*Nedyus quadrimaculatus* (Linnaeus, 1758)	MW886040	**UV**
Curculionidae	Curculioninae	*Anthonomus grandis grandis* Boheman, 1843	XP_050309959	**LW**
Curculionidae	Dryophthorinae	*Rhynchophorus ferrugineus* (Olivier, 1790)	KAF7284001	**LW**
Curculionidae	Lixinae	*Larinus minutus* Gyllenhal, 1835	KY368198	**LW3**
Curculionidae	Lixinae	*Larinus minutus* Gyllenhal, 1835	KY368201	**UV**
Curculionidae	Molytinae	*Hylobius abietis* (Linnaeus, 1758)	MW886096	**LW**
Curculionidae	Scolytinae	*Dendroctonus ponderosae* (Hopkins, 1902)	ENN78452	**LW**
Curculionidae	Scolytinae	*Euwallacea fornicatus* (Eichhoff, 1868)	KY368297	**LW**
Curculionidae	Scolytinae	*Euwallacea fornicatus* (Eichhoff, 1868)	KY368296	**UV**
Curculionidae	Scolytinae	***Ips typographus* (Linnaeus, 1758)**	**Ityp07182**	**LW**
Curculionidae	Scolytinae	***Ips typographus* (Linnaeus, 1758)**	**Ityp12360**	**UV**
Curculionidae	Scolytinae	*Tomicus yunnanensis* Kirkendall & Faccoli, 2008	KY368364	**LW**
Curculionidae	Tychiinae	*Sibinia setosa* (LeConte, 1876)	MW885984	**LW**
Curculionidae	Tychiinae	*Sibinia setosa* (LeConte, 1876)	MW885983	**UV**
Curculionidae	Tychiinae	*Tychius meliloti* Stephens, 1831	MW885986	**LW**
Curculionidae	Tychiinae	*Tychius meliloti* Stephens, 1831	MW885985	**UV1**
Curculionidae	Tychiinae	*Tychius meliloti* Stephens, 1831	MW885987	**UV2**

Type: LW = long wavelength sensitive opsin; **UV** = ultraviolet sensitive opsin.

Alignment of sequences

Three set of opsins were created from sequences listed in Table S1. The first set contained all LW and UV opsins obtained only from Curculionoidea (**CURC-set**), the second group contained solely the orthologous LW opsins of both Curculionoidea and outgroups (Cucujoidea + Chrysomeloidea) (**LWs-set**), and third group included all the UV opsins (**UVs-set**) obtained from Curculionoidea plus outgroups. The CDS sequences of **CURC-set**, **LWs-set** and **UVs-set** were translated into the full-length proteins. These latter were aligned with the software MAFFT (L-INS-I, option) (Katoh et al. 2005) and were generated the three alignments **CURC-set.pro**, **LWs-set.pro** and **UVs-set.pro**. Successively, each set of the protein-coding genes was aligned on the TranslatorX server using as template the alignment obtained for the encoded proteins (Abascal et al. 2010). This web tool ensures that the alignment of DNA sequences is based on the multiple alignment derived from the amino acid translations as a reference and are more accurate (Abascal et al. 2010).

Once downloaded from the TranslatorX server, the DNA multiple alignments were uploaded in MEGA X software (Kumar et al. 2018) to be saved in the various subsets for the tasks described more in details in the next sections.

Assessing the substitution patterns

The compositional heterogeneity of a multiple alignment is a major source of misleading phylogenetic outputs (Kück et al. 2014). The software AliGROOVE was used to test the level of compositional heterogeneity (Kück et al. 2014). AliGROOVE calculates a pairwise mean similarity score between each pair of sequences ($-1 \leq$ range ≤ 1) and returns a matrix of colours. In the matrix, each square is a pairwise comparison between two sequences. The colour varies from deep blue (i.e. non-random similarity, +1) to deep brown (i.e. full random similarity, -1). A red/brown square indicates that heterogeneous aligned positions dominate between the two sequences, while a blue square indicates homogeneous positions (Kück et al. 2014). The AliGROOVE matrices were computed for complete codons, first plus the second positions of each codon, for single positions (first, second and third) and for the translated amino acid sequences.

Estimation of the phylogenetic signal

The amount of phylogenetic signal present in the 18 multiple alignments analysed here (see Results and discussion section) (three codon positions, first two positions, single second codon positions, and amino acids) was evaluated as described here. Firstly, the pairwise-distances among the sequences were computed according to the best fitting evolutionary models selected for the considered alignment (see below the phylogenetic section). Best fitting and more complex evolutionary models account for the multiple substitutions that occur at single position of an alignment thus avoiding the underestimation of the evolutionary process that appends with the simpler models (Nei and Kumar 2000) Then, the distribution of the obtained pairwise distances was analysed in boxplots created with the excel software. Since long time, it is well known that when the distribution of these distances is well above the value 1 there is a substantial loss of phylogenetic signal in in the analysed data set (e.g. Negrisolo et al. 2004).

Phylogenetic analyses

Phylogenetic analyses were based on the Maximum likelihood method. The analyses were performed with the software IQ-TREE 2.3.4 (Minh et al. 2020). The best fitting evolutionary models of the different data sets were selected using the ModelFinder algorithm implemented in IQ-TREE (Kalyaanamoorthy et al. 2017). Partitioned analysis implemented in IQ-TREE was applied to the DNA multiple alignments (Chernomor et al. 2016). Statistical support to the tree topologies were assessed by computing 10,000 replicates of ultrafast bootstrap (UFBoot) (Hoang et al. 2018) and 1000 replicates of the SH-like approximate likelihood ratio test (SH-alrt) (Guindon et al. 2010).

Results and discussion

LW and UV opsins of Ips typographus

The genome search has revealed the presence of two opsin-encoding genes in *I. typographus*. The first gene encodes for a long wavelength absorbing opsin (Ityp07182 LW opsin) containing 377 amino acids with an estimated molecular weight of 41976.29 Daltons and a theoretical pI = 8.68. The second gene encodes for an ultraviolet absorbing opsin (Ityp12360 UV opsin) containing 373 amino acidic residues for a total molecular weight of 42064.25 Daltons and a theoretical pI = 8.13.The tertiary (3D) structures inferred for both Ityp07182 LW and Ityp12360 UV contain 14 α-helices and 2/4 β-sheets and exhibit a high structural similarity (Figure S1).

Twenty-three full-length opsins were identified among beetles of the superfamily Curculionoidea (see below, Table S1). The multiple alignment of these opsins (**CURC-set.pro)** is provided in the Figure S2. Pairwise distances, based on the best fitting evolutionary models (see below), reveal a strong conservation among the opsins of the same type (LW opsins; average-distance = 0.189 ± 0.122. UV opsins, average-distance = 0.317 ± 0.105), with the LW opsins being more conserved than UV opsins (see below, Figure S6B). On the opposite, the distances clearly exceed one (average-distance = 2.889 ± 0.199) when they are computed for pairs of paralogous sequences (i.e. LW vs UV; see below, Figure S6B). In the multiple alignment **CURC-set.pro** the amino acids shared by the whole set of LW and UV opsins are located mainly in the long transmembrane α-helices (Figure S2).

Substitution patterns in the multiple alignments of opsins

The substitution patterns in the multiple alignments obtained from the data sets **CURC-set**, **LWs-set** and **UVs-set** were analysed with AliGROOVE. The results are presented in Figures S3-S5. All DNA multiple alignments (**p123**, **p12**, **p1**, **p2**, **p3**) obtained from **CURC-set** (Figure S3), exhibit a net heterogeneous substitution pattern when the comparisons are made between pairs of paralogous sequences and homogeneous when orthologous sequences are considered. In the case of amino acid multiple alignment (**pro**) the substitution pattern is almost always homogeneous no matter if orthologous or paralogous pairs are compared (Figure S3). The multiple alignments obtained from **LWs-set** and **UVs-set**, which include only orthologous sequences exhibit a very homogenous substitution pattern in both DNA (**p123**, **p12**, **p1**, **p2**) and protein (**pro**) multiple alignments (Figures S4-S5). Third positions (**p3**) of both **LWs-set** and **UVs-set** represent an exception because their multiple alignments exhibit a high level of heterogeneity (Figures S4-S5).

The multiplication of the ancestral opsin into paralogous diverging genes is an old event in insects (Henze and Oakley 2015; Feuda et al. 2016). In our study, the positive (divergent) evolution that characterized the LW and UV opsins of Curculionidea is strongly supported by their very large pairwise distances (Figure S6B). The substitution process was drastically slowed down within each opsin type, as proved by the high degree of protein sequence conservation (Figure S2) and the small distances among orthologous sequences (Figures S6-S8). A pivotal role in the reduction of sequence change was played by structural and functional constraints. Strongly favouring this hypothesis is that only third positions of both the LW and UV opsin coding genes (Figures S6-S8) have experienced multiple substitutions as proved by their very large distances. Most of these changes are synonymous i.e. they do not imply changes of amino acid. However, it should be kept in mind that the comparisons were done with opsins obtained from related beetles. Thus, phylogenetic relatedness could contribute to the sequence stability.

Phylogenetic signal in the multiple alignments of opsins

The 18 multiple alignments that were investigated to evaluate their phylogenetic signal is are listed in Table S2.

Table S2. Multiple alignments tested for phylogenetic signal

Alignment name	Length	Best fitting model	Sequence type
**CURC-set.pro**	394	Q.yeast+F+G4	protein
**CURC-set.p123**	1182	TNe+R4	DNA
**CURC-set.p12**	788	TIM3+F+R3	DNA
**CURC-set.p1**	394	GTR+F+G4	DNA
**CURC-set.p2**	394	TVM+F+G4	DNA
**CURC-set.p3**	394	TNe+I+R3	DNA
**LWs-set.pro**	384	mtZOA+I+R3	protein
**LWs-set.p123**	1152	TNe+I+R3	DNA
**LWs-set.p12**	768	TNe+I+R2	DNA
**LWs-set.p1**	384	TIM2e+G4	DNA
**LWs-set.p2**	384	TVM+F+I+R2	DNA
**LWs-set.p3**	384	TIM2e+I+R2	DNA
**UVs-set.pro**	386	Q.insect+G4	protein
**UVs-set.p123**	1158	TIM3+F+I+G4	DNA
**UVs-set.p12**	772	TPM3u+F+G4	DNA
**UVs-set.p1**	386	GTR+F+G4	DNA
**UVs-set.p2**	386	TPM3u+F+G4	DNA
**UVs-set.p3**	386	TNe+G4	DNA

In Table S2 are listed also the best fitting evolutionary models identified by ModelFinder algorithm that were used to compute the pairwise-distances. The Boxplot relative to pairwise distances computed for the multiple alignments generated with the **CURC-set** (Table S2, Figure S6A) shows a high level of heterogeneity, with **CURC-set.p3** and **CURC-set.p123** exhibiting the maximum range of variation. No multiple alignment presents values constantly below the critical value of 1 (Figure S6A). When, the effect including/excluding paralogous genes/proteins is tested (Figure S6B) it appears immediately evident that pairwise distances higher than 1 are mostly associated to comparisons between paralogous pairs of sequences except for the third positions. Thus, the combination of paralogous sequences at this taxonomic scale (superfamily level) appear clearly detrimental for the phylogenetic signal of the multiple alignments created with species of **CURC-set.**

The boxplots of the pairwise distances computed for the multiple alignments generated with the **LWs-set** and **UVs-set** (Table S2, Figures S7-S8), show patterns of distribution very below the critical value of 1 except for third positions (**p3**), particularly in the **UVs-set.p3** (Figure S8). Obviously, alignments based on whole codons (**p123**) are influenced in their behaviour by the presence of the very variable third positions (Figures S7-S8).

The excess of sequence variability with accumulation of multiple substitutions is very detrimental for the phylogenetic signal present in a multiple alignment. Similarly, a highly heterogeneous substitution pattern is a major source of misleading phylogenetic outputs for the multiple alignments that exhibit it (Kück et al. 2014). However, a multiple alignment with a homogenous substitution pattern is not suitable for phylogenetic purposes if it does not exhibit enough sequence variation. The length of the alignment plays also a role and longer alignments should be preferred over shorter

by the very simple reason that they include more characters. Thus, combining information from AliSCORE and boxplot analyses it appears that the two best multiple alignments showing a homogeneous process of substitution and enough sequence variability are **LWs-set.p12** and **UVs-set.p12**. Other alignments exhibiting good signal are **LWs-set.pro** and **UVs-set.pro**. The **LWs-set.p12** and **UVs-set.p12** multiple alignments were analysed to investigate the phylogenetic positions of LW and UV opsins of *I. typographus.*

Phylogenetic analyses

Trees obtained from the phylogenetic analyses performed on **LWs-set.p12** and **UVs-set.p12** are presented in the Figure 3 (see main text). Both trees were created by applying a partitioning scheme (Chernomor et al. 2016). In particular, the best model for **LWs-set.p12** resulted to be TIM3e+G4:part1 (i.e. first positions), TVM+F+I+R2:part2 (i.e. second positions). In the case of **UVs-set.p12** the best partition scheme was TIM2e+G4: part1 (i.e. first positions), TPM3u+F+R2: part2 (i.e. second positions).

The phylogenetic relationships among the opsin sequences in the trees obtained from both **LWs-set.p12** and **UVs-set.p12** are in good agreement with the taxonomic placement of the species from what they were obtained with some exceptions (e.g. the placement of *Agnesiotis pilosula* LW). In the tree obtained from **LWs-set.p12** most of the nodes and branches received statistical corroboration (Figure 3A). The LW sequence of *I. typographus* is grouped with the sequence of *Euwallacea fornicatus* LW and *Rhynchophorus ferrugineus* LW. This type of relationship was recovered also in the analysis performed on **LWs-set.pro** multiple alignment (Figure S9). Most of nodes and branches of the tree obtained from **UVs-set.p12** multiple alignment receive statistical support. In this tree the UV sequence of *I. typographus* is clustered with the UV sequence of *E. fornicatus* and this relationship receive statistical corroboration. The same relationship is found also in the tree obtained from **UVs-set.pro** (Figure S10). Like in the LW tree, there is good agreement with opsins relationships and taxonomic placement of the species.

The multiplication of the ancestral opsin into paralogous diverging genes and protein products is an old event in insects (Henze and Oakley 2015; Feuda et al. 2016). In our study, the divergent evolution that characterized also the LW and UV opsins of Curculionidea is strongly supported by their very large pairwise distances (Figure S6B). The substitution process was drastically slowed down within each opsin type, as proved by the high degree of sequence conservation (Figure S2) and the small distances among orthologous sequences (Figures S6-S8). A pivotal role in the reduction of sequence change was played by structural and functional constraints. Strongly favouring this hypothesis is that only third positions of both the LW and UV opsin coding genes (Figures S6-S8) have experienced multiple substitutions as proved by their very large distances. Most of these changes are synonymous i.e. they do not imply changes of amino acid. However, it should be kept in mind that the comparisons were done with opsins obtained from related beetles. Thus, phylogenetic relatedness could contribute partly to the sequence stability.

References

Abascal F, Zardoya R, Telford MJ (2010). TranslatorX: multiple alignment of nucleotide sequences guided by amino acid translations. Nucleic Acids Res 38:W7-W13. https://doi.org/10.1093/nar/gkq291

Altschul SF, Gish W, Miller W et al (1990). Basic local alignment search tool. J Mol Biol 215:403–410. https://doi.org/10.1016/S0022-2836(05)80360-2

Chernomor O, von Haeseler A, Minh BQ (2016). Terrace aware data structure for phylogenomic inference from supermatrices. Syst Biol 65:997–1008. https://doi.org/10.1093/sysbio/syw037

Duvaud S, Gabella C, Lisacek F et al (2021). Expasy, the Swiss Bioinformatics Resource Portal, as designed by its users. Nucleic Acids Res 49:W216–W227. https://doi.org/10.1093/nar/gkab225

Feuda R, Marletaz F, Bentley MA, Holland PW (2016). Conservation, duplication, and divergence of five opsin genes in insect evolution. Genome Biol Evol 8:579–587. https://doi.org/10.1093/gbe/evw015

Guignard Q, Allison JD, Slippers B (2022) The evolution of insect visual opsin genes with specific consideration of the influence of ocelli and life history traits. BMC Ecol Evol 22:2. https://doi.org/10.1186/s12862-022-01960-8

Guindon S, Dufayard J-F, Lefort V et al (2010). New algorithms and methods to estimate maximum-likelihood phylogenies: assessing the performance of PhyML 3.0. Syst Biol 59:307–321. https://doi.org/10.1093/sysbio/syq010

Henze MJ, Oakley TH (2015). The dynamic evolutionary history of pancrustacean eyes and opsins. Integr Comp Biol 55:830–842. https://doi.org/10.1093/icb/icv100

Hoang DT, Chernomor O, von Haeseler A et al (2018). UFBoot2: Improving the ultrafast bootstrap approximation. Mol Biol Evol 35:518–522. https://doi.org/10.1093/molbev/msx281

Kalyaanamoorthy S., Minh BQ, Wong TKF et al (2017). ModelFinder: Fast model selection for accurate phylogenetic estimates. Nat Methods 14:587–589. https://doi.org/10.1038/nmeth.4285

Katoh K, Kuma K, Toh H, Miyata T (2005). MAFFT version 5: improvement in accuracy of multiple sequence alignment. Nucleic Acids Res 33:511–518. https://doi.org/10.1093/nar/gki198

Kück P, Meid SA, Gross C et al (2014) AliGROOVE – visualization of heterogeneous sequence divergence within multiple sequence alignments and detection of inflated branch support. *BMC Bioinformatics* 15, 294. http://www.biomedcentral.com/1471-2105/15/294

Kumar S, Stecher G, Li M et al (2018). MEGA X: Molecular Evolutionary Genetics Analysis across computing platforms. Mol Biol Evol 35:1547–1549. https://doi.org/10.1093/molbev/msy096

McCulloch KJ, Macias-Muñoz A, Briscoe AD (2022). Insect opsins and evodevo: what have we learned in 25 years? Philos Trans R Soc Lond B 377:20210288. https://doi.org/10.1098/rstb.2021.0288

Minh BQ, Schmidt HA, Chernomor O et al (2020). IQ-TREE 2: New models and efficient methods for phylogenetic inference in the genomic era. Mol Biol Evol 37:1530–1534. https://doi.org/10.1093/molbev/msaa015

Negrisolo E, Minelli A, Valle G (2004). The Mitochondrial genome of the house centipede Scutigera and the monophyly versus paraphyly of myriapods. Mol Biol Evol 21:770–780. https://doi.org/10.1093/molbev/msh078

Nei S, Kumar K (2000) *Molecular evolution and phylogenetics* Oxford University Press, New York.

Powell D, Groβe-Wilde E, Krokene P et al (2021). A highly-contiguous genome assembly of the Eurasian spruce bark beetle, *Ips typographus*, provides insight into a major forest pest. Commun Biol 4:1059. https://doi.org/10.1038/s42003-021-02602-3

Sharkey CR, Fujimoto MS, Lord NP et al (2017). Overcoming the loss of blue sensitivity through opsin duplication in the largest animal group, beetles. Sci Rep 7:8. https://doi.org/10.1038/s41598-017-00061-7

Sharkey CR, Powell GS, Bybee SM (2021) Opsin evolution in flower-visiting beetles. Front Ecol Evol 9:676369. https://doi.org/10.3389/fevo.2021.676369

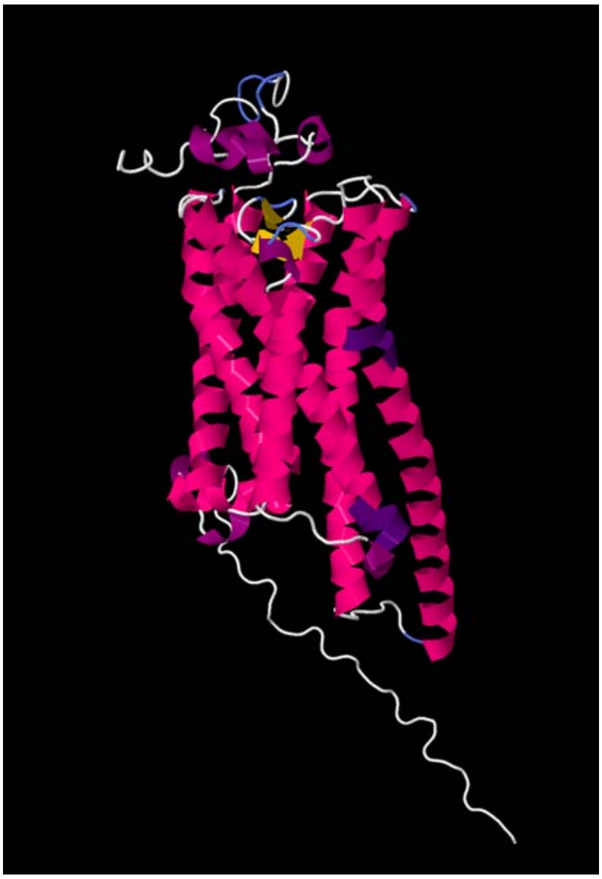

Predicted 3D structure of
***Ips typographus* Ityp07182 LW**

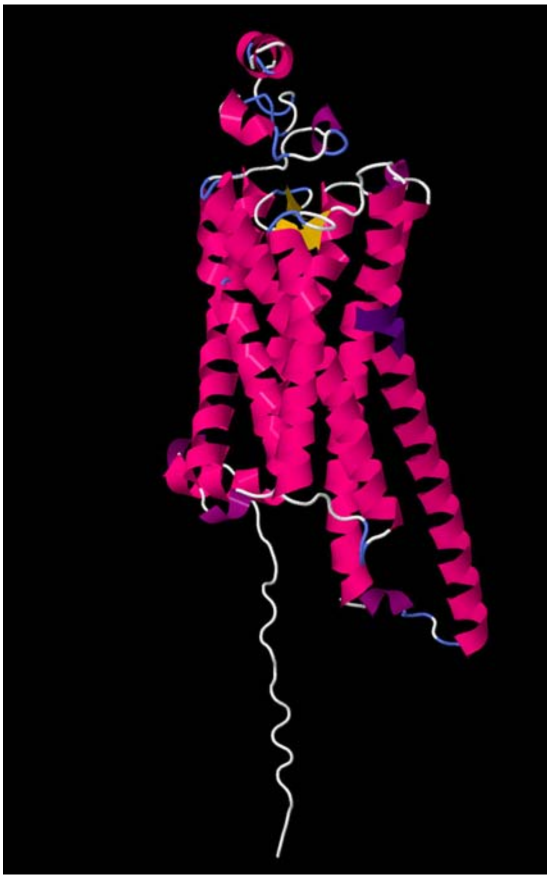

Predicted 3D structure of
***Ips typographus* Ityp12360 UV**

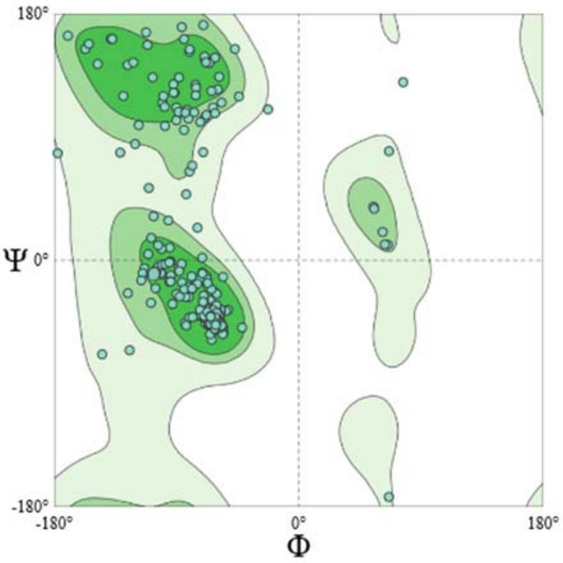

Ramachandran plot for the 3D structure of *Ips typographus* Ityp07182 LW

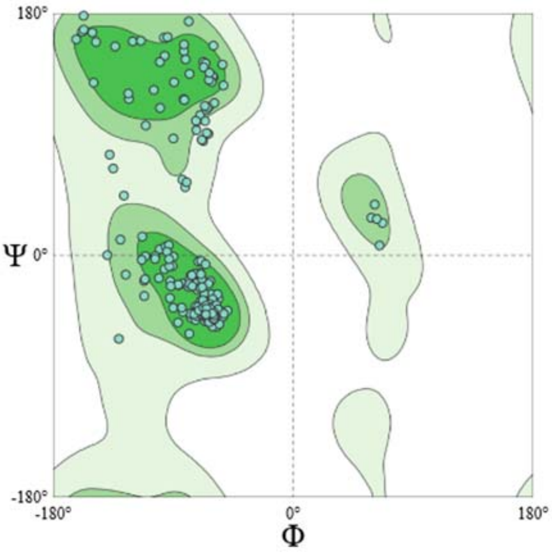

Ramachandran plot for the 3D structure of *Ips typographus* Ityp12360 UV

Figure S1. Predicted 3D structures of the *Ips typographus* Ityp07182 LW and Ityp12360 UV

On top the 3D stuctures figured with the JMOL software. On bottom the Ramachandran plots obtained for the 3D structure from the homology modelling process.

Figure S2. 1/2

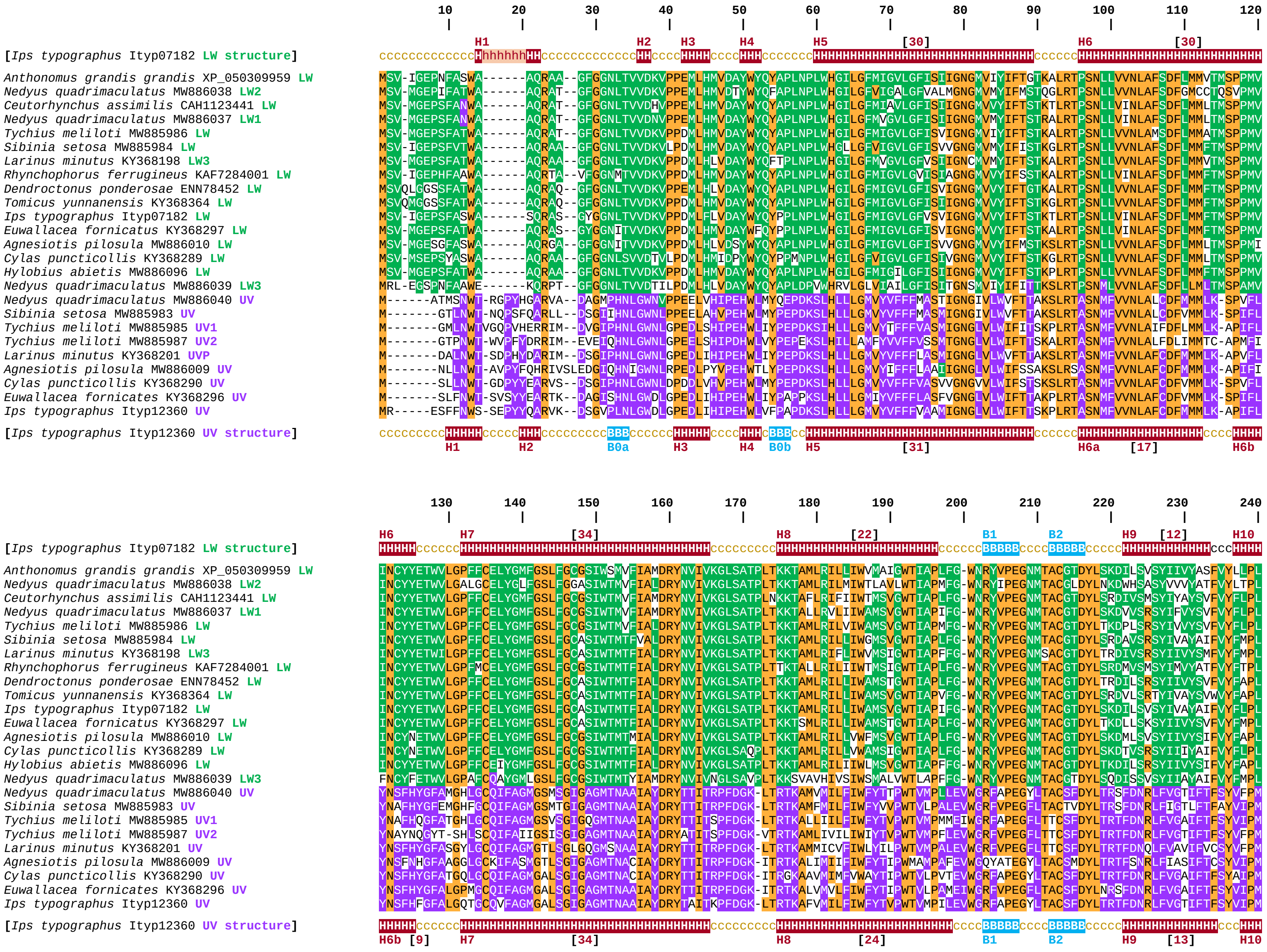
[Ips typographus Ityp07182 LW structure]
Anthonomus grandis grandis XP_050309959 LW
Nedyus quadrimaculatus MW886038 LW2
Ceutorhynchus assimilis CAH1123441 LW
Nedyus quadrimaculatus MW886037 LW1
Tychius meliloti MW885986 LW
Sibinia setosa MW885984 LW
Larinus minutus KY368198 LW3
Rhynchophorus ferrugineus KAF7284001 LW
Dendroctonus ponderosae ENN78452 LW
Tomicus yunnanensis KY368364 LW
Ips typographus Ityp07182 LW
Euwallacea fornicatus KY368297 LW
Agnesiotis pilosula MW886010 LW
Cylas puncticollis KY368289 LW
Hylobius abietis MW886096 LW
Nedyus quadrimaculatus MW886039 LW3
Nedyus quadrimaculatus MW886040 UV
Sibinia setosa MW885983 UV
Tychius meliloti MW885985 UV1
Tychius meliloti MW885987 UV2
Larinus minutus KY368201 UVP
Agnesiotis pilosula MW886009 UV
Cylas puncticollis KY368290 UV
Euwallacea fornicates KY368296 UV
Ips typographus Ityp12360 UV
[Ips typographus Ityp12360 UV structure]
H1 H2 B0a H3 H4 B0b H5 [31] H6a [17] H6b
H6 H7 [34] H8 [22] B1 B2 H9 [12] H10
H6b [9] H7 [34] H8 [24] B1 B2 H9 [13] H10

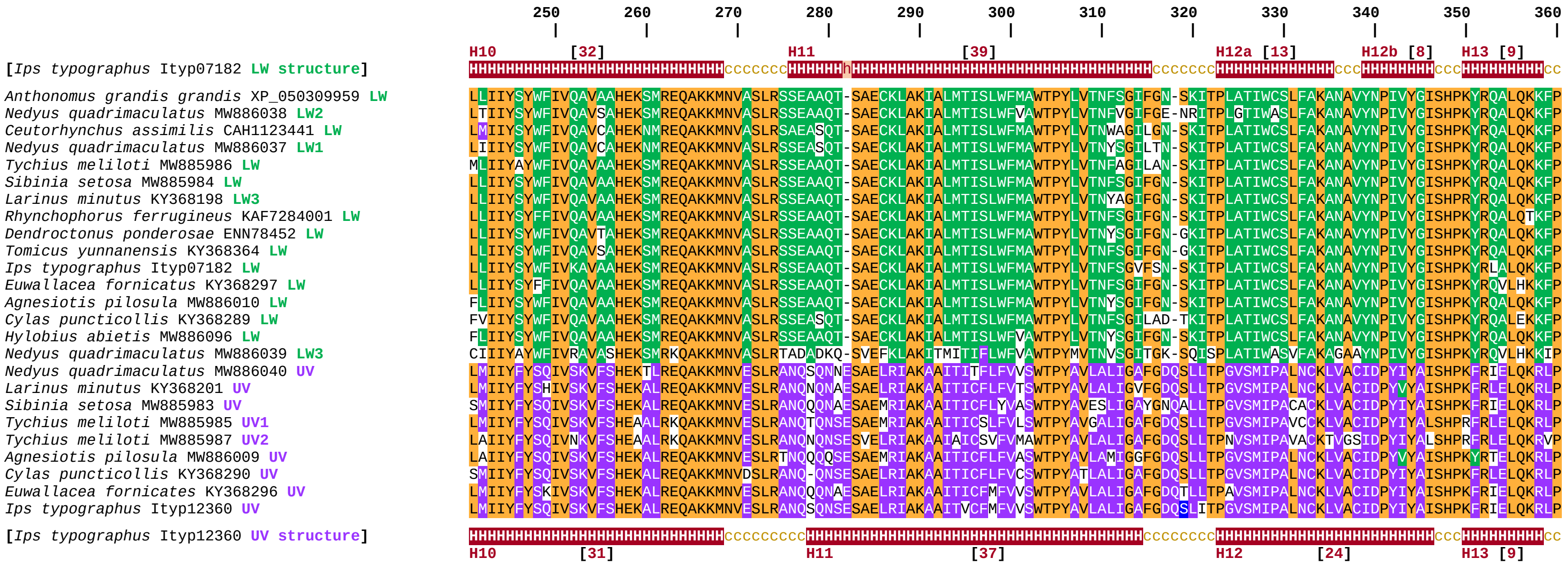

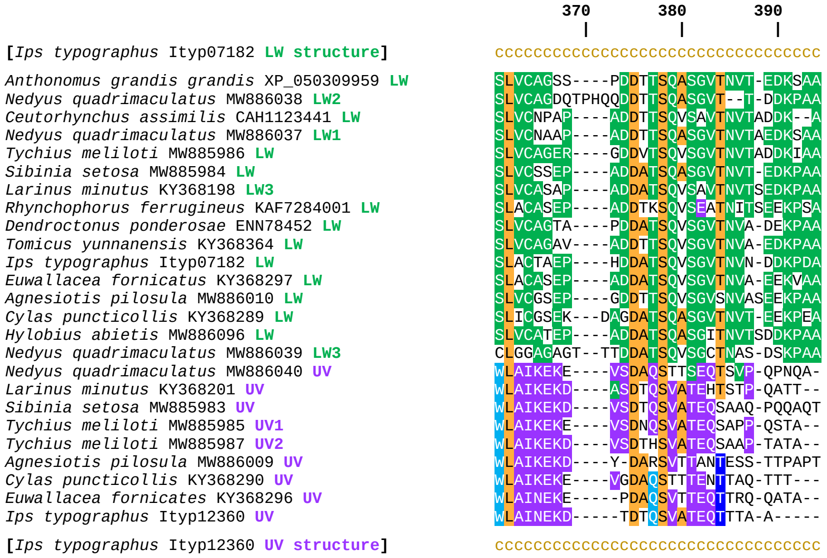

Figure S2. Multiple alignment of the CURC-set.pro, generated with MAFFT (L-INS-I, option).
X, amino acid conserved in LW and UV opsins. X, amino acid conserved in LW opsins; X, amino acid conserved in UV opsins. C, random coil. H, alpha helix, B, beta sheet. Number in brackets, number of amino acids forming the helix.
H1-H13, number of alpha helix, B0-B4, number of beta sheet.
Structures based on homology modelling (see main text and Figure S1).

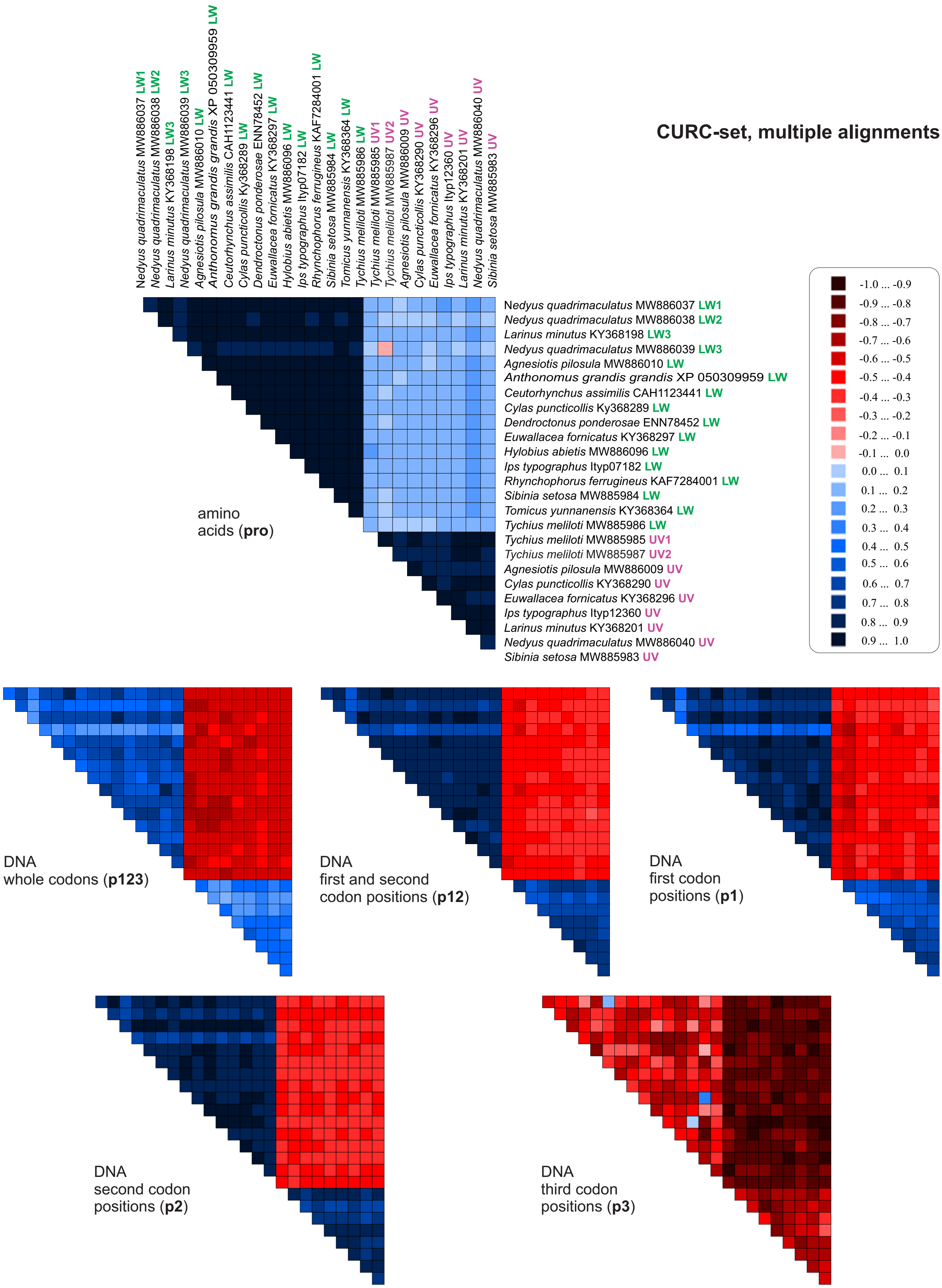

Figure S3. AliGROOVE matrices for CURC-set multliple alignments.

The AliGROOVE matrix is provided for every single multiple alignment (see main text for details). AliGROOVE calculates a pairwise mean similarity score between each pair of sequences (-1 ≤ range ≤ 1), and return a coloured matrix. In the matrix, each pairwise comparison is represented by a coloured square, which varies from deep blue (i.e. non-random similarity, +1) to deep-brown (i.e. full random similarity, deep-brown coloured; -1). A red square indicates that heterogeneous aligned positions dominate between the two sequences, while a blue square indicate that the opposite is true.

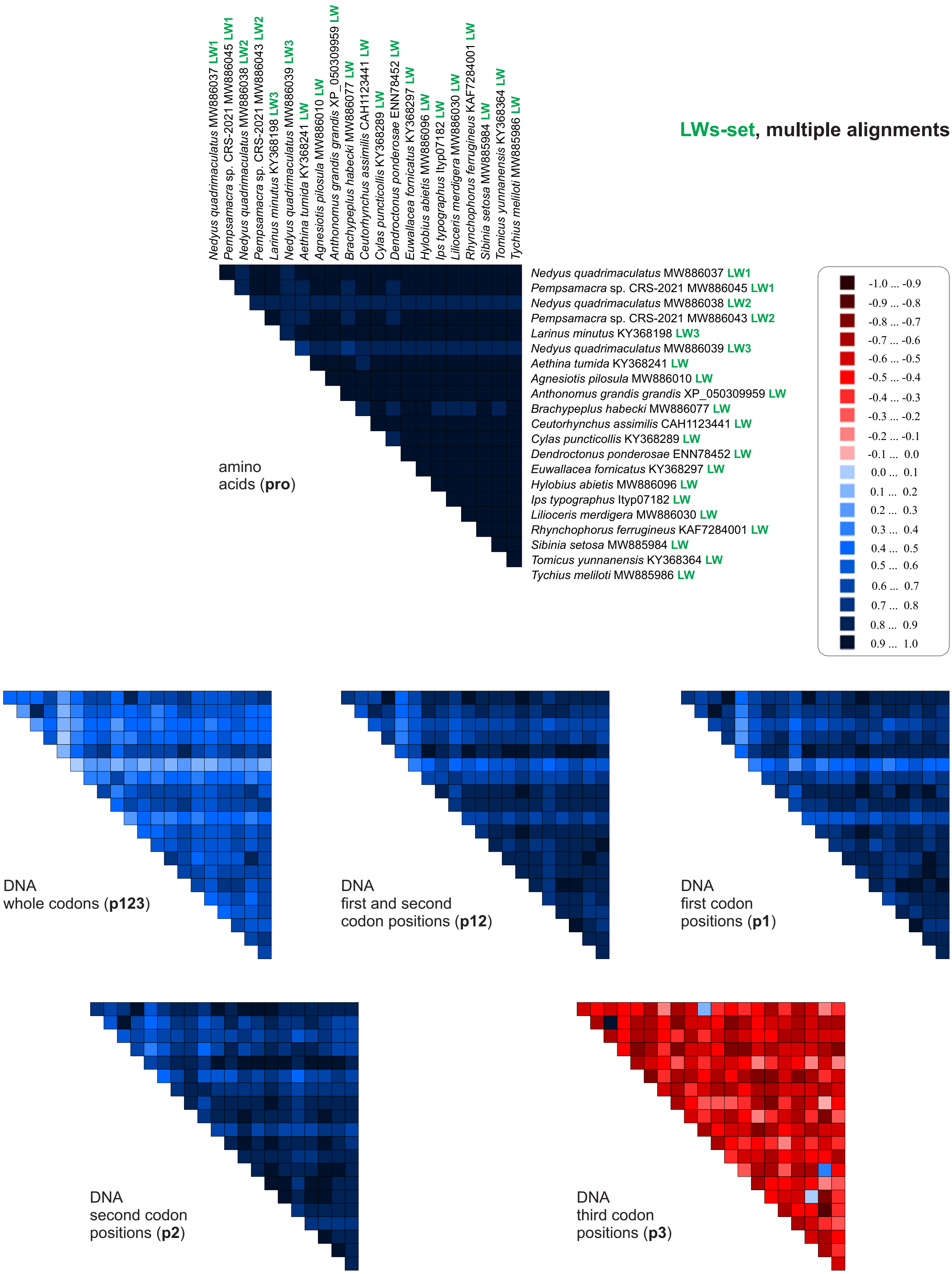

Figure S4. AliGROOVE matrices for Ws-set multliple alignments.

The AliGROOVE matrix is provided for every single multiple alignment (see main text for details). AliGROOVE calculates a pairwise mean similarity score between each pair of sequences (-1 ≤ range ≤ 1), and return a coloured matrix. In the matrix, each pairwise comparison is represented by a coloured square, which varies from deep blue (i.e. non-random similarity, +1) to deep-brown (i.e. full random similarity, deep-brown coloured; -1). A red square indicates that heterogeneous aligned positions dominate between the two sequences, while a blue square indicate that the opposite is true.

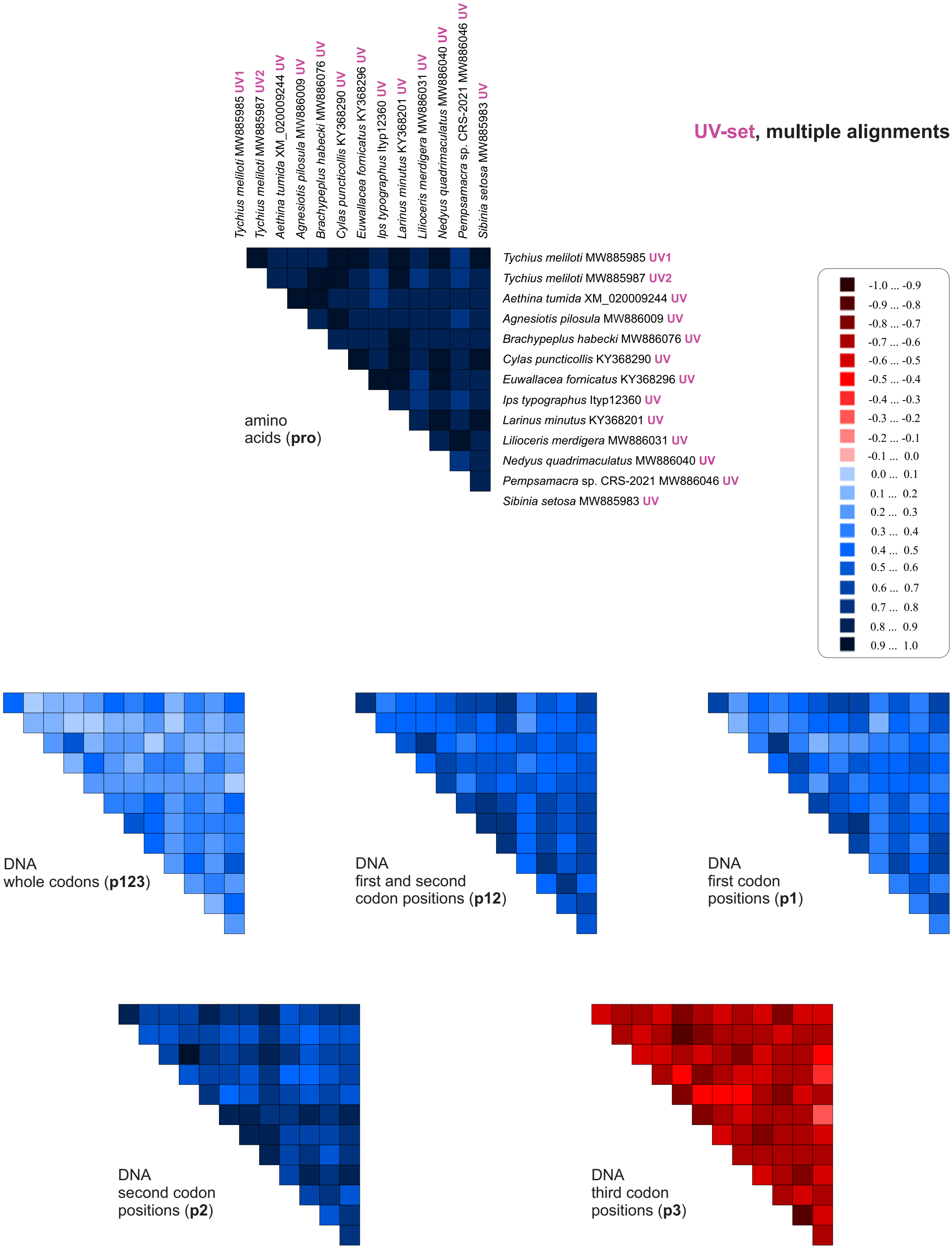

Figure S5. AliGROOVE matrices for UV-set multliple alignments.

The AliGROOVE matrix is provided for every single multiple alignment (see main text for details). AliGROOVE calculates a pairwise mean similarity score between each pair of sequences ($-1 \leq range \leq 1$), and return a coloured matrix. In the matrix, each pairwise comparison is represented by a coloured square, which varies from deep blue (i.e. non-random similarity, +1) to deep-brown (i.e. full random similarity, deep-brown coloured; -1). A red square indicates that heterogeneous aligned positions dominate between the two sequences, while a blue square indicate that the opposite is true.

CURC-set, multiple alignments

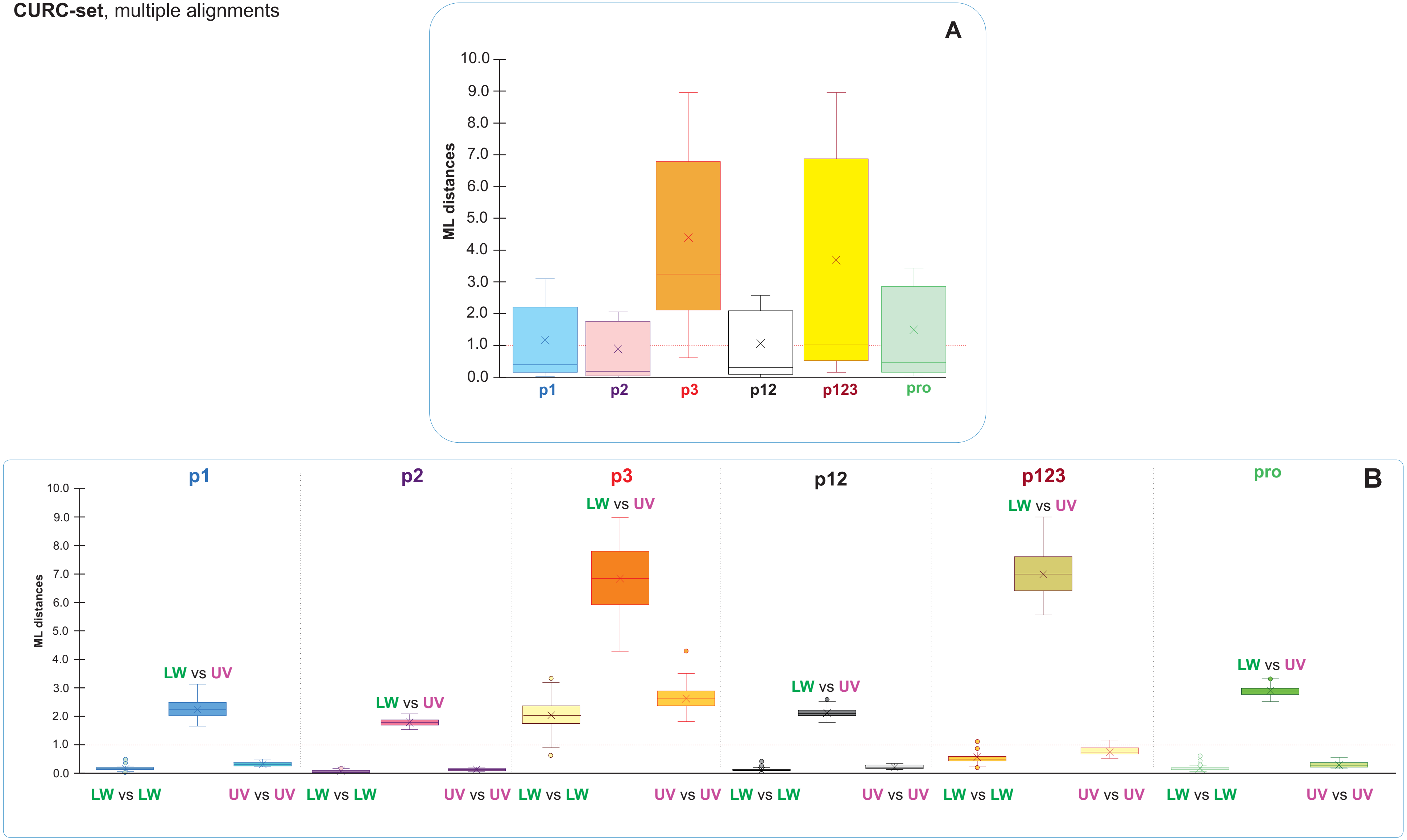

Figure S6. Boxplot for CURC-set multiple alignments.

A. Boxplot of the pairwise distances computed for the **p1, p2, p3, p12, p12, p123** and **pro** multiple alignments of the **CURC-set**. All pairwise-distances are included.

B. Boxplot of the pairwise distances computed for the **p1, p2, p3, p12, p12, p123** and **pro** multiple alignments of the **CURC-set**. Pairwise-distances are split into three groups for each alignment i.e. **LW** vs **LW**, **LW vs UV** , and **UV vs UV** for understanding the effect of including/excluding paralogous opsins. Middle line, median value; x, mean; box upper and lower edges, interquartile range, including 50% of the observations; circles, outliers. Whisker lines extend for 1.5 times the interquartile range.

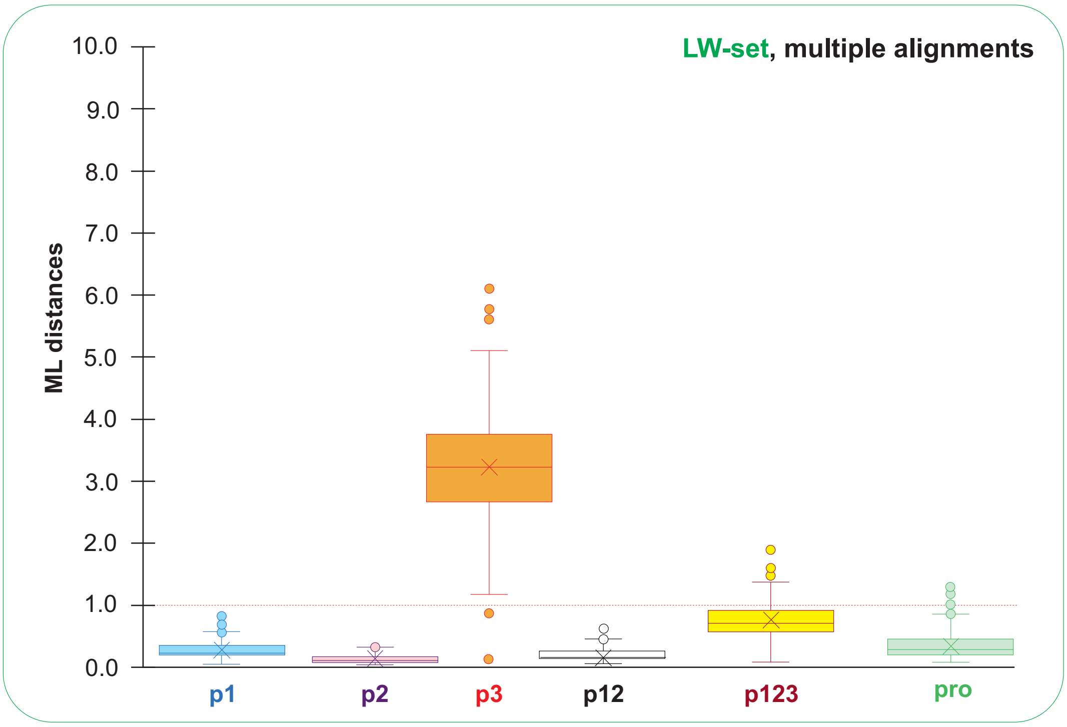

Figure S7. Boxplot fo LW-set, multiple alignments

Boxplot of the pair-wise distances computed for the **p1, p2, p3, p12, p123**, and **pro** multiple alignments of the LW-set. Middle line, median value; x, mean; box upper and lower edges, interquartile range, including 50% of the observations; circles, outliers. Whisker lines extend for 1.5 times the interquartile range.

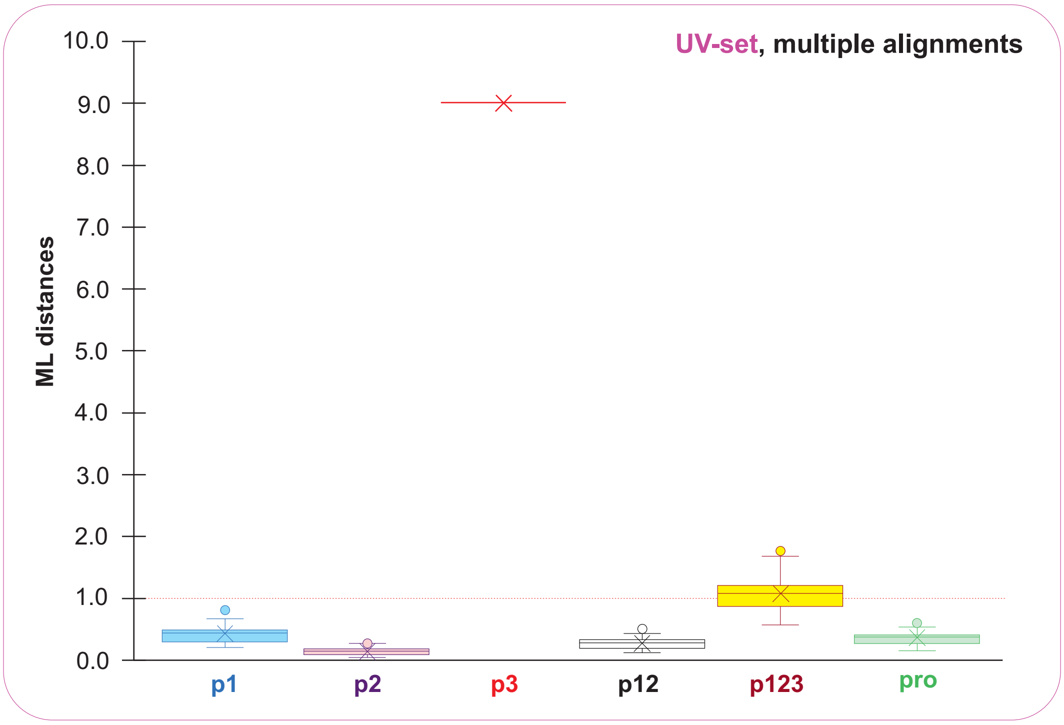

Figure S8. Boxplot fo UV-set, multiple alignments

Boxplot of the pair-wise distances computed for the **p1, p2, p3, p12, p123**, and **pro** multiple alignments of the UV-set. Middle line, median value; x, mean; box upper and lower edges, interquartile range, including 50% of the observations; circles, outliers.Whisker lines extend for 1.5 times the interquartile range.

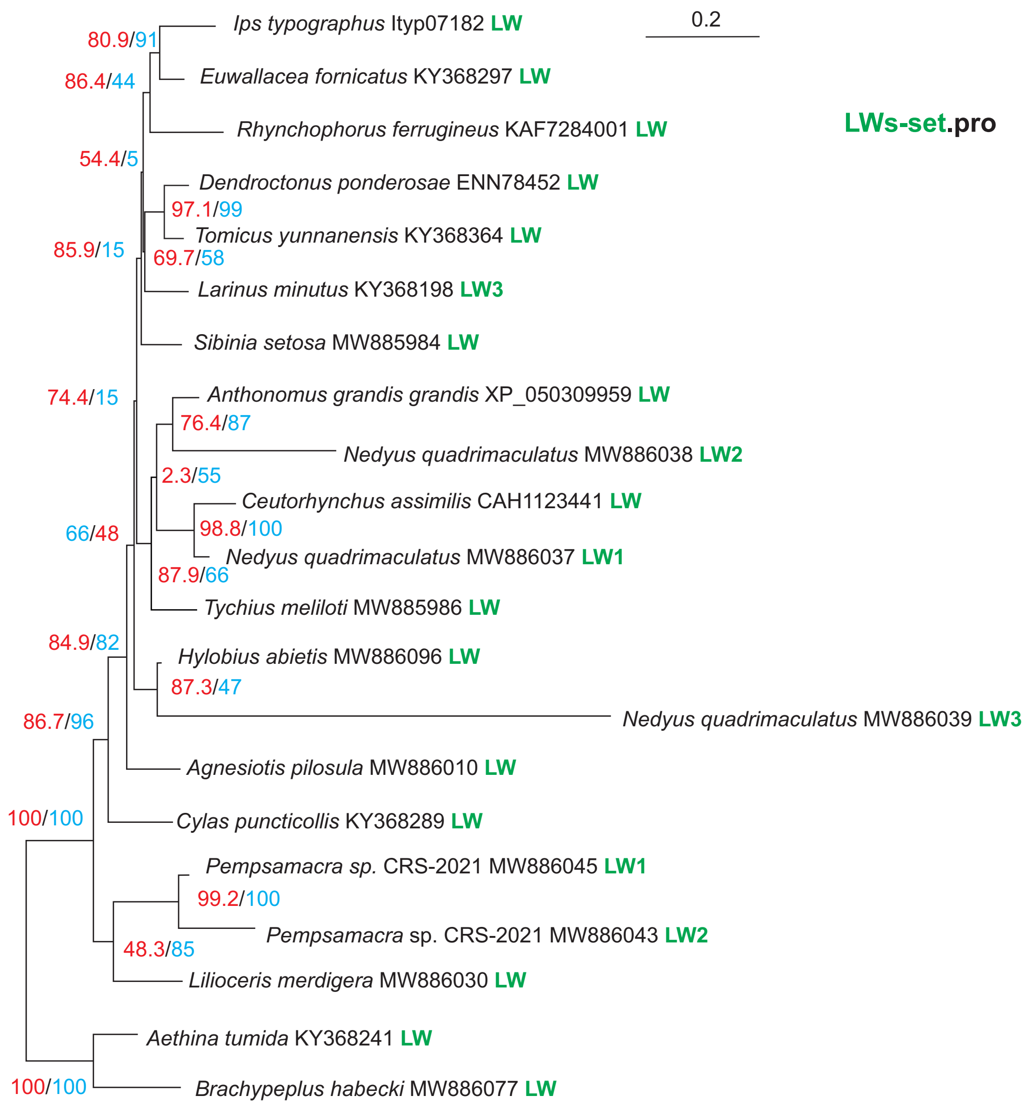

Figure S9. Maximum likelihood tree based on LWs-set.pro

Maximum likelihood phylogram (-log = 4547.2567) obtained with IQ-TREE by applying the best fitting evolutionary model mtZOA+I+R3. Scale bar represents number of substitutions per site.

UFBoot/SH-alrt expressed in percentage.

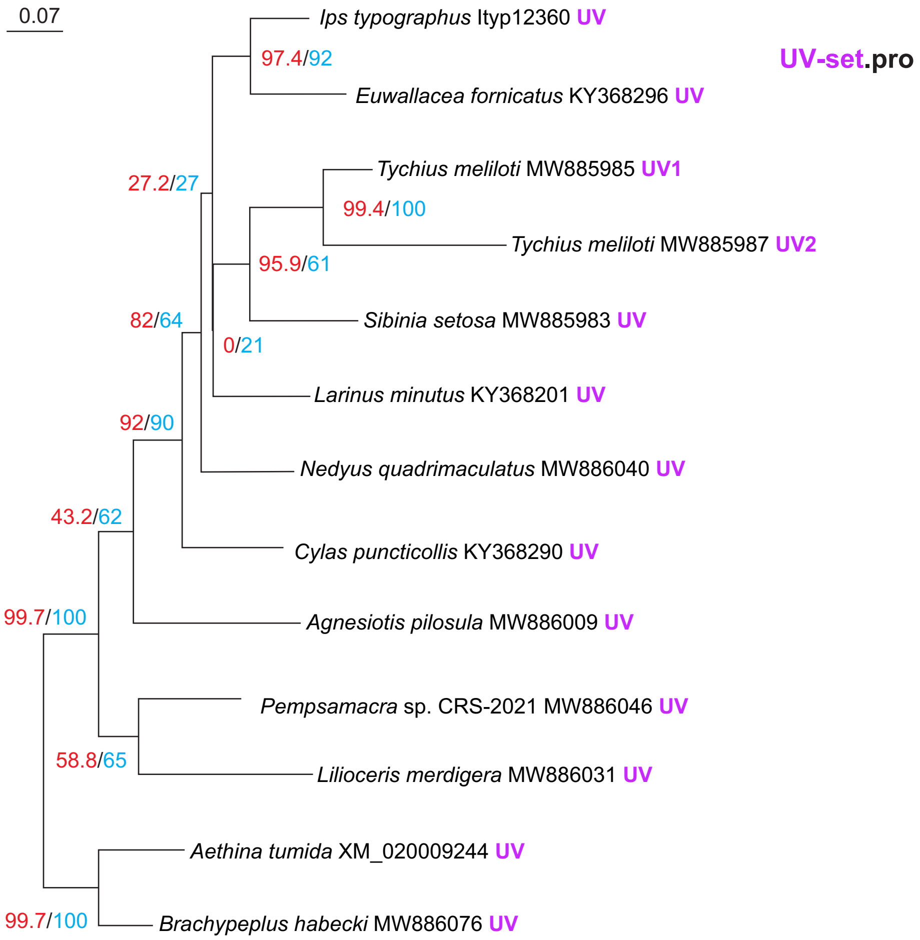

Figure S10. Maximum likelihood tree based on UV-set.pro

Maximum likelihood phylogram (-log = 4308.3637) obtained with IQ-TREE by applying the best fitting evolutionary model Q.insect+G4. Scale bar represents number of substitutions per site.

UFBoot/SH-alrt expressed in percentage.
