# Supplementary material for: Ips typographus vision system: a comprehensive study
Source: J Comp Physiol A Neuroethol Sens Neural Behav Physiol. 2024 Sep 27;211(1):101–12. doi: 10.1007/s00359-024-01717-2 (PMC11846742; doi:10.1007/s00359-024-01717-2)
Supplement: Supplementary file 2 — Supplementary Material 2 [file 359_2024_1717_MOESM2_ESM.docx]

**Supplementary material**

Document on Molecular evolution of opsins containing Table S1 and S2, Figures S1-S10


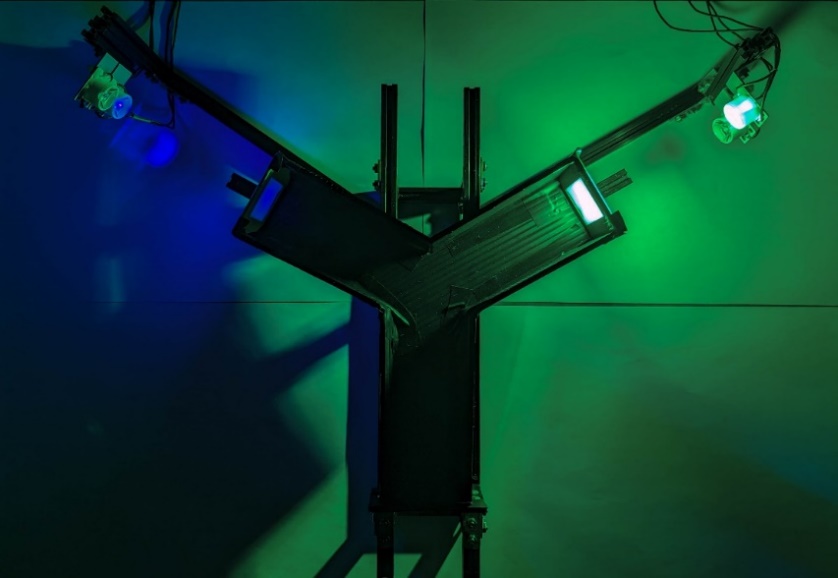


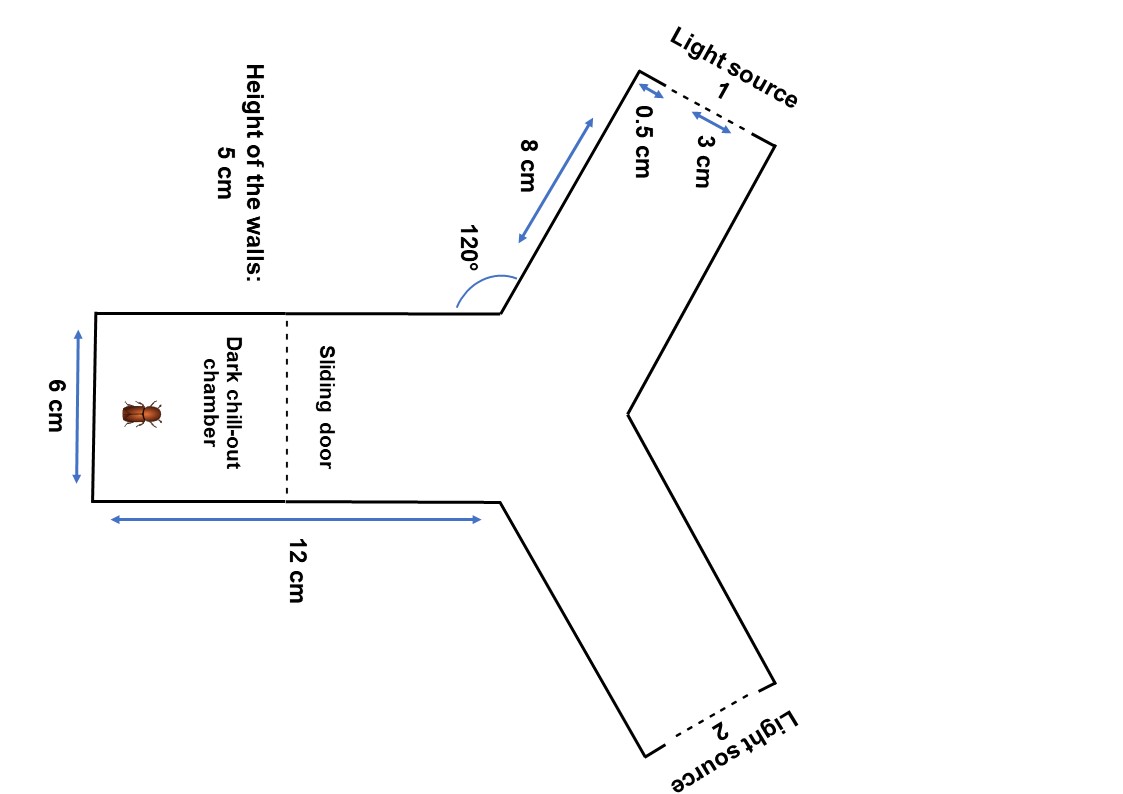


Figure S11. Scheme of the Y-maze device (up) and a photo of the device operating with UV LED light on the upper arm and green LED light on the lower arm (down).
